# A Review of Theoretical Studies on Carbon Monoxide Hydrogenation via Fischer–Tropsch Synthesis over Transition Metals

**DOI:** 10.3390/molecules28186525

**Published:** 2023-09-08

**Authors:** Maryam Jamaati, Mostafa Torkashvand, Saeedeh Sarabadani Tafreshi, Nora H. de Leeuw

**Affiliations:** 1 Department of Physics, Iran University of Science and Technology, Narmak, Tehran 16846-13114, Iran; 2 Department of Chemistry, Amirkabir University of Technology (Tehran Polytechnic), No. 350, Hafez Avenue, Tehran 15916-34311, Iran; 3 School of Chemistry, University of Leeds, Leeds LS2 9JT, UK; 4 Department of Earth Sciences, Utrecht University, 3584 CB Utrecht, The Netherlands

**Keywords:** Fischer-Tropsch synthesis, DFT, carbon monoxide, transition metals, cobalt

## Abstract

The increasing demand for clean fuels and sustainable products has attracted much interest in the development of active and selective catalysts for CO conversion to desirable products. This review maps the theoretical progress of the different facets of most commercial catalysts, including Co, Fe, Ni, Rh, and Ru. All relevant elementary steps involving CO dissociation and hydrogenation and their dependence on surface structure, surface coverage, temperature, and pressure are considered. The dominant Fischer–Tropsch synthesis mechanism is also explored, including the sensitivity to the structure of H-assisted CO dissociation and direct CO dissociation. Low-coordinated step sites are shown to enhance catalytic activity and suppress methane formation. The hydrogen adsorption and CO dissociation mechanisms are highly dependent on the surface coverage, in which hydrogen adsorption increases, and the CO insertion mechanism becomes more favorable at high coverages. It is revealed that the chain-growth probability and product selectivity are affected by the type of catalyst and its structure as well as the applied temperature and pressure.

## 1. Introduction

Current human life is highly dependent on energy sources from fossil fuels, which consequently produce significant amounts of carbon dioxide and carbon monoxide [1]. Hence, achieving clean, low-carbon valuable liquid fuels and chemicals (e.g., methanol, formaldehyde, and methane, in addition to other hydrocarbons) through CO/CO_2_ conversion is of paramount importance and the topic of much research, both in industry and academia [1,2,3]. 

Fischer–Tropsch synthesis (FTS) is an attractive but complex technology, which aims to convert synthesis gas (a mixture of carbon monoxide and hydrogen) into a wide range of hydrocarbons and oxygenates [4,5,6,7,8,9,10,11]. It is known that FTS takes place through the dissociative adsorption of CO and H_2_, followed by hydrogenation, to result in the generation of CH_x_ (x = 1–3) intermediates and C + C coupling reactions [12]. Hence, the FTS process includes the following steps: the adsorption of the surface species, CO activation, C−O bond dissociation, CH_x_ formation, chain growth, and desorption of H_2_O [13,14]. The chosen catalyst plays a vital role in FTS [15]

CO activation is the first step in FTS, which occurs via either a direct or H-assisted pathway. As such, two distinct mechanisms exist for the chain growth in the FTS process, i.e., the carbide mechanism, proposed by Fischer and Tropsch [16], and CO insertion, proposed by Pichler and Schulz [14,17,18,19]. In the carbide mechanism, CO is activated and dissociates after adsorption on the catalyst surface. The produced C_1_ species then undergo sequential hydrogenation to form CH_x_, which acts as the chain initiator and propagator for long-chain hydrocarbon products or contributes to the chain growth through C–C coupling reactions [14,19,20]. Note, however, that the CO insertion mechanism is more complicated and less well-known than the carbide mechanism, since a number of oxygenating intermediates are produced during the process [18]. In this process, the insertion of adsorbed CO into a growing hydrocarbon and C−O bond scission leads to the formation of CH_x_O, which is followed by the removal of adsorbed O [14,19,20,21]. It is worth noting that the formation of C or CH_x_ species from CO dissociation is initially required for chain propagation in both the insertion and carbide mechanisms [14,19]. The active dissociation happens through direct CO or H-assisted CO dissociation, according to whether the adsorption state H is involved or not, i.e., whether the C–O bond breaks directly into C and O or breaks after HCO (or COH) production from CO hydrogenation [6]. 

A variety of products is obtained from CO hydrogenation in the Fischer–Tropsch process. Hence, enhancing the efficiency of FTS toward desirable products, such as gasoline or olefin, requires catalysts with high chain-growth probability but low selectivity toward methane [3,18,22,23]. The aforementioned reactions proceed at the active sites of common FT catalyst surfaces including Co and Fe, which are successfully industrially utilized [2,22,24,25,26,27,28]. Although other metals, such as nickel and ruthenium, exhibit similar activity toward short-chain (Ni) or long-chain (Ru) hydrocarbon formation, their poor product selectivity and limited availability make them undesirable [9,25,29]. Cobalt, a highly favorable catalyst that possesses high activity and selectivity toward the production of long-chain hydrocarbons from syngas [24,30,31], also shows slow deactivation [24], low water gas shift (WGS) activity [32], and high stability [4,6,19,33]. Furthermore, industry uses a Co catalyst for its resistance to the carbon deposition problem [21,34]. A comparison between iron and cobalt catalysts involves a trade-off between desired resistance to deactivation (Co is more resistant than Fe), selectivity (Fe has a tendency to produce products such as olefins and oxygenated compounds, while Co is more selective toward long-chain hydrocarbon formation) [3], price (Fe is over 200 times cheaper than Co) [9], working temperature (Co works at relatively lower temperature than Fe) [3], and pressure (up to 40 bar for Co compared to 20 bar for Fe) [35]. However, the activity per unit catalyst mass and its lifetime show cobalt to be an expensive catalyst material [36]. One strategy to overcome this issue is to use a nanoparticulate form of Co with higher activity, even though its activity and selectivity remarkably vary by size [36]. The formation of a large amount of carbide on Fe during the reactions [37] and the activity of Fe toward the water−gas shift reaction (WGSR) [13] reduce its catalytic activity, which is undesirable. To mitigate these difficulties, much research focused on identifying FTS catalysts to replace or improve the performance of Fe and Co catalysts [27,38], for example, by alloying these metals with others [22,38] or via the addition of noble metal promoters [39].

Ruthenium is the most active catalyst at the lowest reaction temperature; it does not need a promoter and is suitable for long-chain hydrocarbon formation [13], where the sufficiently high rate of CO dissociation increases the rate of the chain growth. However, its application is limited owing to its low abundance and resulting high cost [40]. Nickel-based catalysts are highly active toward hydrogenation reactions and very promising single-stage WGS [41] with high selectivity toward methane, which is, however, not commercially valuable [13]. Furthermore, its usage as a promoter in Co does not significantly influence the selectivity toward higher hydrocarbons [4], although it was shown that its alloying with cobalt and iron may enhance the catalytic activity and the chain-growth probability [23,38]. Rh adsorbs CO in both molecular and dissociated forms [42] and shows the significant formation of attractive ethanol and other oxygenates [40], where the CO dissociation rate on different catalyst surfaces results in a range of products. Since CO cannot dissociate on transition metals such as Cu, Pd, Ir, and Pt, methanol is their main product, and they are, therefore, not as attractive as catalysts [40]. 

Previous review articles extensively explored the catalytic Fischer–Tropsch synthesis process. Shiba et al. [43] conducted a comprehensive review focusing on the production of lower olefins over cobalt catalysts via Fischer–Tropsch synthesis. Their study highlighted the key factors influencing olefin selectivity and discussed the underlying mechanisms. Rommens et al. [44] provided a molecular-level perspective on Fischer–Tropsch synthesis, elucidating the various steps and intermediates involved in the conversion of syngas to hydrocarbons. Their review incorporated recent advancements in theoretical and experimental studies, offering valuable insights into the reaction pathways and surface reactions. Rytter et al. [45] explored the deactivation and regeneration processes of commercial-type Fischer–Tropsch cobalt catalysts. Their study investigated the causes of catalyst deactivation and proposed strategies for catalyst regeneration, with a focus on enhancing catalyst stability and activity. Chai et al. [46] presented a mechanistic understanding of Fischer–Tropsch synthesis over Fe-carbide catalysts. Their review elucidated the reaction mechanisms, surface species, and active sites involved in the conversion of syngas to hydrocarbons over Fe-C catalysts, shedding light on the unique catalytic properties and selectivity of these systems. Furthermore, Mousavi et al. [47] developed a generalized kinetic model for iron- and cobalt-based Fischer–Tropsch synthesis catalysts. Their study aimed to describe and predict the catalytic performance of these catalysts under various operating conditions, providing a valuable tool for process optimization and catalyst design. In addition to these review articles, several other notable studies contributed to the understanding of Fischer–Tropsch synthesis. Qi et al. [6] conducted a comprehensive investigation on Co-based Fischer–Tropsch catalysis using transient kinetic studies and theoretical models. Their work provided valuable insights into the reaction kinetics, catalyst deactivation, and the influence of various factors on the catalytic performance of Co-based catalysts. Horacek [48] focused on the effect of promoters, catalyst support, and reaction conditions in the selection of catalysts for Fischer–Tropsch synthesis. Their study encompassed the impact of different promoters, such as noble metals or alkali metals, on the activity, selectivity, and stability of Fischer–Tropsch catalysts. These reviews played a crucial role in summarizing the currently existing knowledge, identifying research gaps, and guiding further investigations in the field. However, to the best of our knowledge, there is still the need for a comprehensive review specifically addressing the hydrogenation of carbon monoxide. As such, the present study aims to fill this gap by providing a thorough analysis of transition metals through theoretical studies.

It is noteworthy that, due to the vital importance of CO conversion to clean fuels and useful materials and the complexity of FTS involving hundreds of elementary reactions on catalytic surfaces, many efforts were devoted to identifying the catalyst nature and developing the related technology. Moreover, understanding the effects of flat versus defective surfaces as well as surface coverage gives insight into product selectivity, which is highly desirable in a variety of industrial applications. Additionally, it is of crucial importance to know how temperature and pressure affect the activity and selectivity of catalysts during the FTS process. Hence, in the present review paper, we aim to summarize the results of the preferred mechanism governing the CO conversion, selectivity, and activity of relevant catalyst surfaces; the interaction of intermediates with the surface; the plausible ways of the chain growth; O removal; and active adsorption sites, especially on cobalt-based catalyst surfaces. The paper is organized as follows: in Section 2, we demonstrate the results obtained from a wider investigation of cobalt-based catalysts, and, in Section 3, we give brief summaries of non-cobalt catalyst achievements. 

## 2. Cobalt-Based Catalysts

### 2.1. Cobalt

In the pioneering work by Hovi et al., CO hydrogenation on cobalt model catalysts was studied using Monte Carlo simulations [49]. Their general results showed that the termination of carbon chains is the rate-limiting reaction step, so the chain growth is slow; that the vacant adsorption sites do not significantly affect the selectivity or the activity of the catalyst; that CO dissociation requires overcoming a low barrier; and that water removal during the FT process is fast. In addition, they suggested a negative pressure dependence according to the shift of the catalyst selectivity toward C_2_ products rather than methane. Therefore, further theoretical investigation is necessary to guide the tailoring of Co surfaces toward the CO hydrogenation mechanism, reaction, and activation energy and the selectivity toward different products. 

**Figure 1:**
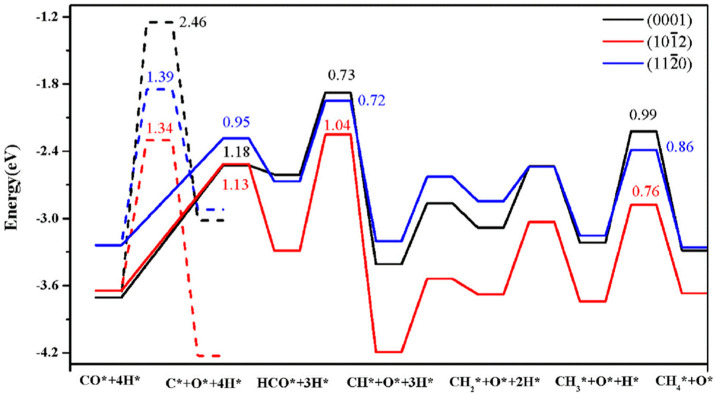
Reaction energy diagram for CO methanation on Co (0001), ($10\bar{1}2$), and ($11\bar{2}0$) surfaces [21].

The CO dissociation or H-assisted hydrogenation on flat surfaces such as Co (0001), Co ($10\bar{1}2$), Co ($11\bar{2}0$), Co (11$\bar{2}$1), and stepped Co (11$\bar{2}$1) were studied by DFT calculations. The reaction of CO and H_2_ to hydrocarbons was studied by Santos-Carballal et al. on Co (11$\bar{2}$1) [13]. The calculated reaction energies are listed in Table 1, Table 2 and Table 3, and the reaction coordinates are depicted in Figure 1. The discrepancy in reaction energies is associated with the different binding strengths of the intermediates. Endothermic values indicate pseudo-equilibrated steps during the FT process. The more exothermic reactions coupled with the lower barriers for CO dissociation on Co ($10\bar{1}2$) and Co ($11\bar{2}0$) make them both thermodynamically and kinetically more favorable than the Co (0001) surface [21]. The remarkable variation in the barriers and reaction energies in the work of Liu et al. [21] (Table 1, Table 2 and Table 3) indicated that direct CO dissociation is highly sensitive to the structure with respect to H-assisted dissociation and CH_x_ hydrogenation. Furthermore, open Co ($10\bar{1}2$) and ($11\bar{2}0$) surfaces are highly active in the direct CO dissociation, owing to a favorable fourfold binding site and the lack of competition between dissociated carbon and oxygen. However, the contribution of adsorbed atomic hydrogen with low sensitivity during the activation process makes CH_x_ hydrogenation the least sensitive to the surface structure. In contrast, the H-assisted CO activation pathway is more favorable than the direct CO activation pathway on the Co (0001) surface, which shows that the sensitivity to the structure of CO activation leads to methane as the product, especially on open surfaces. The dominance of hydrogen-assisted CO dissociation as a CO activation pathway was likewise confirmed by Yang et al. [7]. Their study illustrated the importance of the hydrogen concentration on the surface to determine the reactivity of adsorbed CO and methane formation. At low H concentrations and high CO pressures, CHOH decomposition followed by CH hydrogenation is the pathway to methane formation. Torkashvand et al. used DFT methods to study FTS [50], identifying adsorption energies, reaction energies, and reaction barriers for methane formation by the hydrogenation of CO over Co (0001).

**Figure 2:**
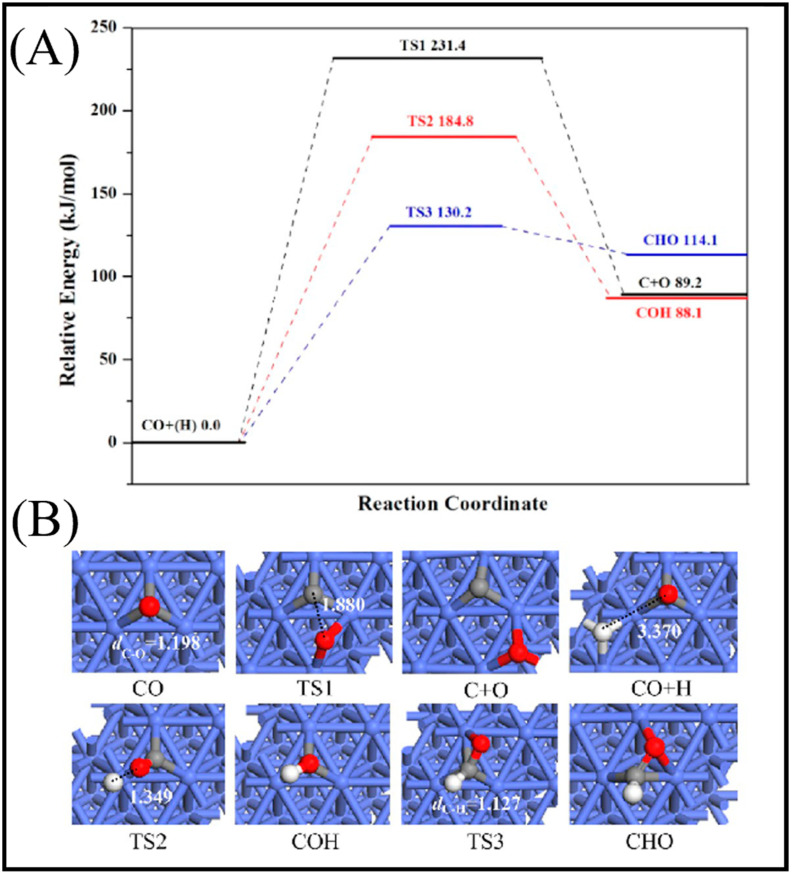
(**A**) Reaction energy diagram of CO dissociation and hydrogenation and (**B**) corresponding initial, transition, and final state structures. Bond lengths are in Å [14].

**Figure 3:**
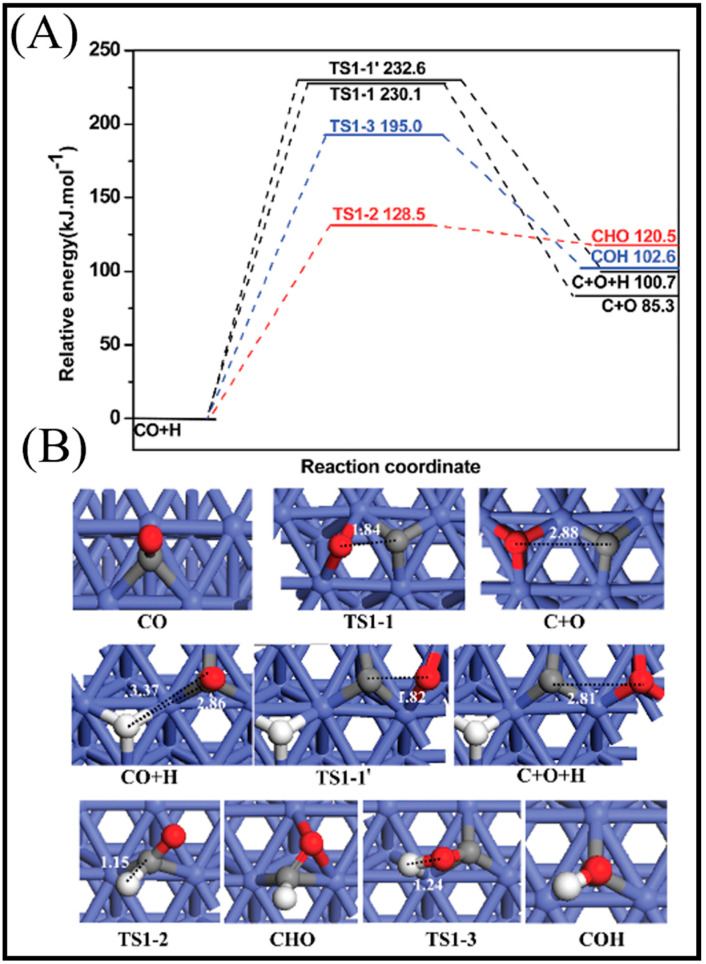
(**A**) The potential energy diagram for CO activation at 500 K and (**B**) the related states, from initial to final, of CO activation. Bond lengths are depicted in Å [51].

As described in Table 2 [14], most of the reactions in CH_x_ formation are endothermic, while most of the C−C chain formation reactions via C(H)O insertion into CH_x_ are exothermic. Figure 2 indicates the direct dissociation of CO with two alternative COH and CHO hydrogenation routes. Between the two pathways, the CHO intermediate is the dominant CO activation pathway over the Co (11$\bar{2}$1) surface, where CH hydrogenation is more favorable than its dissociation due to a much lower activation barrier. CH_4_ can be easily produced through hydrogenation, and only CH_3_ hydrogenation is the rate-determining step during the CH_4_ formation process. In summary, the preferred pathway for CH_4_ formation is via CH + 3H → CH_2_ + 2H → CH_3_ + H → CH_4_. It was also found that the most feasible C−C chain formation is through CHO insertion into CH_x_ (x = 1, 2), rather than CO insertion. It should be noted that the C−O bond must cleave for the further hydrogenation of CH_x_CH_y_O intermediates to take place, whereas CH_x_CH_y_ hydrogenates through different possible hydrogenation routes. CH_2_-CH_2_ coupling is the most favorable process versus the least favorable CH_3_-CH_3_ coupling among the CH_x_CH_y_ reactions. Furthermore, CHO and CH_2_CH formation are the rate-determining steps in the complete reaction and C_2_ hydrocarbon formation, respectively. Hence, improving their formation leads to the promotion of C_2_ hydrocarbon formation. Additionally, the rate-determining steps of C_2_ hydrocarbon formation were compared on the flat and stepped Co (11$\bar{2}$1) surfaces (Table 3). Step sites facilitate the formation of CHO and CH_2_CH while suppressing CH_4_ formation. The initiation, growth, and termination mechanisms of C–C chain formation are also considered on the Co (0001) surface [51]. Adsorption indicates that H_2_ dissociation to the H atom is favorable, and the adsorbed H atom interacts with other species to achieve the chain growth. It is found that the catalyst activity toward direct CO dissociation is very low, and the major route of CH_x_ (x = 1–3) formation begins from the CHO intermediate during CO and H co-adsorption (see Figure 3). CHO is a key intermediate, with a dissociation into CH that is preferred over its hydrogenation to CHOH and CH_2_O. According to the reaction energies, the formation of CH_x_ (x = 1–3) and, especially, the formation of CH are easier than that of CH_3_OH, and the dominant pathway is H-assisted CO dissociation. Further studies showed that the hydrogenation of CH and CO/CHO insertion into CH are more favorable than its dissociation and coupling, whereas CH_2_ prefers to dissociate into CH_2_ or undergo CHO insertion to produce a CH_2_CHO intermediate. As for CH_3_, even if its formation is facile, it is simply dissociated into CH_2_ intermediates. The first elementary step in CO conversion is CO dissociation, which may happen through the carbide or insertion mechanism, and there is debate in the literature whether the direct or H-assisted pathways govern the initial CO dissociation, with some authors accepting that both mechanisms occur. Valero and Raybaud provided an overview of FT reaction mechanisms on cobalt-based catalysts [35]. Although no agreement exists between the results presented by researchers, all studies agreed that the starting reaction strongly depends on the surface structure and active sites. Furthermore, the adsorption energies and surface coverages are affected by temperature and the reactant pressure. In the case of flat Co catalysts, authors claimed that the insertion mechanism is the most feasible, while the carbide mechanism is dominant on stepped surfaces. 

The elementary steps from C_2_ to C_6_ and the α-olefin selectivity through the hydrogenation and dehydrogenation of n-alkyl groups at the Co (0001) surface were investigated in the work by Cheng et al. [52]. They illustrated that the barriers to hydrogenation and dehydrogenation reactions and the chemisorption energies of α-olefins are similar for all chain species. They also found that the chain-length dependence of the paraffin/olefin ratio originated from the chain-length dependence of the van der Waals interaction. Furthermore, they observed an abnormal ethane/ethylene ratio, which is related to the greater chemisorption energy of ethylene. The almost equal distance of the unsaturated C atoms in the n-alkyl groups (*n* = 2–6) of the three nearest Co atoms suggested the similar bonding of these species, owing to their similar chemisorption energies. CH_3_ chemisorbs with a higher energy than the other species, and the C–Co distances in CH_3_ are smaller than those of the other hydrocarbons in the n-alkyl groups (*n* = 2–6). This fact is attributed to the repulsive interactions between the alkyl groups and the surface in the n-alkyl groups (*n* = 2–6), which are greater than the interaction between H and the surface in CH_3_. 

**Figure 4:**
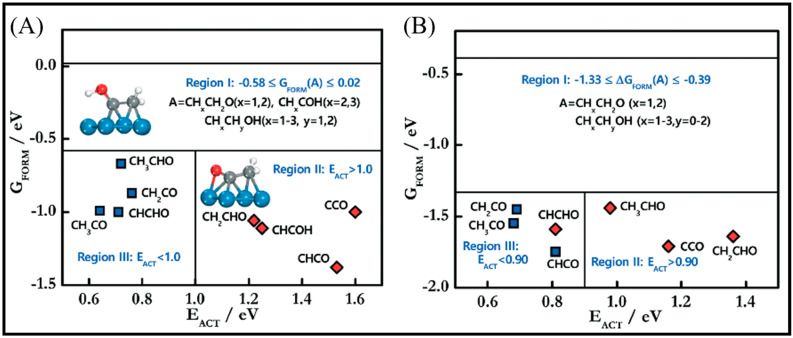
Formation free energies GFORM of C_2_-oxygenate intermediates as a function of their activation energies for C–O bond scission on (**A**) Co (0001) and (**B**) stepped Co [18].

Sensitivity to the structure in the chain growth and selectivity are of crucial importance to control CO conversion production. The highly efficient FT process needs a high probability of the chain growth with the low selectivity toward methane [18]. Su et al. focused on the chain growth and CO insertion at active sites on the Co (0001), stepped Co, and Co ($10\bar{1}1$) surfaces [18] and also considered the coverage effects on the chain-growth mechanism and selectivity. Figure 4 shows the formation free energies and activation energies (E_ACT_) of C–O bond scission for 19 C_2_-oxygenate intermediates. As can be seen, four intermediates with a much smaller E_ACT_, which have minimum energy pathways via the CO insertion mechanism, are placed in region III. The CO insertion mechanism on the Co (0001) and stepped Co with C−O bond scission for five C_2_-oxygenate intermediates are represented in Figure 4A. As is clear, the CO insertion of CH leads to two different pathways: the CO insertion of CH followed by hydrogenation and the CH_2_ formed by CH hydrogenation. An additional pathway (shown as the black line) is comprised of the sequential addition of hydrogen to CH, which leads to CH_3_ production with small activation energy barriers. As such, it can be concluded that CH_x_ + H is more favorable than CH_x_ + CO (x = 1,2) on the Co (0001) and stepped Co surfaces, and all three pathways can play roles in the CO insertion on the stepped Co. The energy profile of the chain growth (CO insertion (red line) versus the carbide (black line)) and methanation (blue line) mechanisms on (A) Co (0001), (B) stepped Co, and (C) Co ($10\bar{1}1$) surfaces, depicted in Figure 4B, shows that adsorbed intermediates from methanation (CH_2_ and CH_3_) more strongly bind than the adsorbed intermediates from the CO insertion (CHCO and CHCHO) and carbide mechanisms (CH and HCO). As such, the bond strength of the methanation is the highest, while the bond strength of the carbide mechanism is the lowest, and the carbide mechanism is more difficult to achieve than the methanation and CO insertion mechanisms. 

Furthermore, the CO conversion and hydrogenation on the Co (111) surface for the production of desired products, ethane (C_2_H_6_) and other hydrocarbons as opposed to methane (CH_4_), was considered by Santos-Carballal and co-workers [13]. The adsorption of single CO molecules at four distinct positions of the Co (111) surface showed variation in the structural, vibrational, electronic, and thermodynamic properties of the surface, originating from the charge transfer from the surface to the π* antibonding molecular orbitals of the adsorbate, and they inferred that single molecule adsorption positively affects the stability of the surface. The interaction of a single H_2_ molecule with the Co (11$\bar{2}$1) surface was also explored as well as CO, and the following results were obtained: the adsorption energies of H_2_ are also negative but are less favorable than the interaction between CO and the Co (11$\bar{2}$1) surface. Due to the short radius of H, its average binding distance to the surface is smaller than that of CO. Although H atoms are negatively charged because of the charge transfer from the surface, it is lower in comparison to that of the CO molecule. 

**Figure 5:**
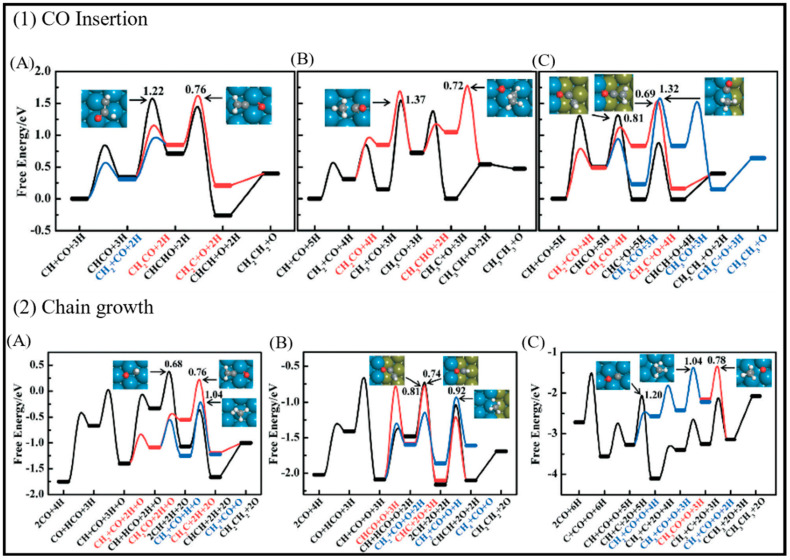
(**Panel 1**), the energy profiles of the CO insertion mechanism by the C–O bond scission in (**A**) CHCHO and CH_2_CO and (**B**) CH_3_CO and CH_3_CHO on Co (0001), and (**C**) CHxCO (x = 1–3) on stepped Co. The reference zero of the energy scale corresponds to the energy of adsorbed CH, CO and H. (**Panel 2**), The energy profiles of chain growth (CO insertion (red line) vs. carbide (black line)) and methanation (blue line) mechanisms on (**A**) Co (0001), (**B**) stepped Co and (**C**) Co ($10\bar{1}1$). The reference zero of the energy scale corresponds to the free energy of CO and H_2_ in the gas phase. [18].

Finally, it was found that the adsorption of a single H_2_ molecule makes the surface more catalytically reactive. The minimum energy pathways for the co-adsorption of CO and H on the Co (11$\bar{2}$1) surface via a number of intermediates are presented in Figure 5(1A). Co-adsorption is favorable and, after its occurrence, both the CO and H molecules remain in their initial adsorption sites, although just further from the surface. Although the direct pathway contains a saddle point, the co-adsorption of CO and H provides enough energy to overcome this high energy point. OH and C are the final generated products from both the direct and indirect pathways. It is understood that the coexistence of large coverages of H with a number of C_1_ intermediates on the Co (11$\bar{2}$1) surface leads to the possibility of three different pathways [13]. In the molecular pathway, the C_2_H_2_ molecule can undergo a further reduction to form the olefin molecule (C_2_H_4_). The calculation results indicate that the intermediates of the dissociated pathways are less stable, which suggests that the additional CH can produce C_2_H_6_ formation. CH_4_ formation from two methyl (CH_3_) groups makes unstable intermediates with high energies. Hence, from the reaction profiles proposed here, one can deduce that the Co (11$\bar{2}$1) surface has the largest selectivity toward C_2_H_6_.

The methanation from CO hydrogenation on stepped and terrace cobalt surfaces was explored as well [53]. C–O bond breaking in direct CO dissociation was found to be more feasible as the main pathway on the stepped surface. The most favorable H-assisted CO dissociation pathways on Co ($11\bar{2}1$) have lower barriers than on the Co (0001) surface, owing to the important contribution of step–edge sites to the CO dissociation process. As such, the formation of CH_x_ through a direct CO scission takes place on the step–edge sites. However, methane is produced on both step–edge and terrace sites, where the final hydrogenation step of CH_3_ to CH_4_ is the rate-controlling step on both types of surfaces. More hydrogenation causes “extra-methane” formation, in which the methanation is favored due to variation in the composition of the surface adsorbates. Thus, lower coverages of CH_x_ decrease the adsorption energy of CO and, consequently, lower the rate of CO desorption. Very recently, Zijlstra et al. [54] also examined the step–edge effect on the CO activation and chain growth on terrace Co (0001) and step–edge Co ($11\bar{2}1$) surfaces, with the majority of the hydrogenation reactions shown to be endothermic. The hydrogenation of adsorbed C–C species on the surfaces revealed the preference for ethylene product over ethane, and the C_2_ coupling products on the Co (0001) surface are more stable than the C_3_ products, although there is no significant difference on the stepped Co ($11\bar{2}1$) surface, and paraffin is less likely to form than olefin. Consequently, their extrapolation data for C_4+_ formation barriers did not show a clear stability difference between hydrocarbon chains with four or more C atoms and C_3_ species. Moreover, simulations unveiled the high activity of the step–edge-containing Co ($11\bar{2}1$) surface in the FT reaction, whereas the addition of terrace Co (0001) sites in a dual-site increased CH_4_ selectivity. From the obtained kinetic data [54], one can conclude the following results: as CO partial pressure increases, the CH_4_ selectivity in addition to the CO conversion and turnover frequency values drop. The reduction in the CH_4_ selectivity is related to higher CO coverages as a result of rising pressures. In addition, increasing the H_2_ partial pressure causes slightly lower CO_2_ selectivity and a remarkable decrease in chain-growth probability. In summary, they found that the carbide mechanism dominates owing to the lower barriers for CO dissociation and CH_x_ + CH_y_ coupling, as opposed to CO insertion.

**Figure 6:**
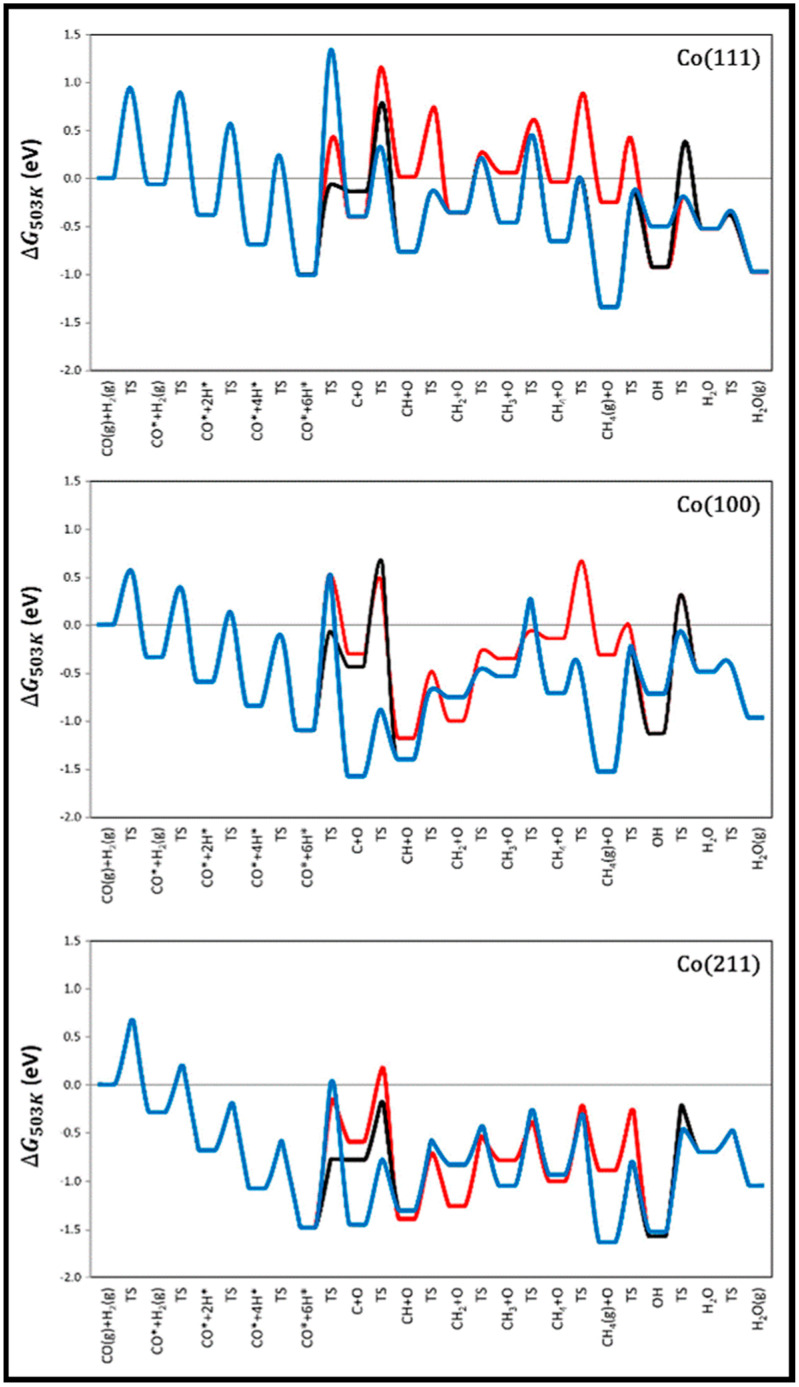
Free energy surface diagrams for methanation on (**top**) site A/Co (111), (**middle**) site B/Co (100), and (**bottom**) site C/Co (211). Blue curvature shows methanation via direct CO dissociation and OH disproportionation; black color indicates CO scission via the HCO intermediate and OH hydrogenation route; red color presents pathway via the COH intermediate [55].

The methanation at three different sites, the cobalt surfaces of Co (11$\bar{2}$1) and Co (10$\bar{1}$0) and the step sites represented by Co (21$\bar{3}$1)), was also examined by the Van Helden group [55]. Figure 6 displays the free energy surface diagrams for methanation at 503 K on site A/Co (11$\bar{2}$1), Site B/Co (10$\bar{1}$0), and site C/Co (21$\bar{3}$1), which indicate that (i) a very high barrier of CO fracture governs the slow rate on site A, and the water formation via the OH disproportionation is almost easy; (ii) the overall rate on site B is controlled by the CO scission, which includes a combination of direct dissociation and both the HCO and COH H-assisted ones; (iii) water formation via the OH disproportionation process is, nevertheless, easy on the B site, where OH formation is the rate-limiting step causing O removal mostly from CO_2_ formation, rather than H_2_O formation; (iv) CO dissociation at the C site proceeds through an HCO intermediate, which needs to consume more CO than on site B, and water formation easily occurs via an OH disproportionation process; and (v) since the energy profile of the methanation process is relatively flat on the C site, a very high rate of methane formation is obtained. Moreover, not only one site contributes to the reaction network, but the intermediates formed at one site can migrate to another site and, hence, undergo different reaction steps. 

**Figure 7:**
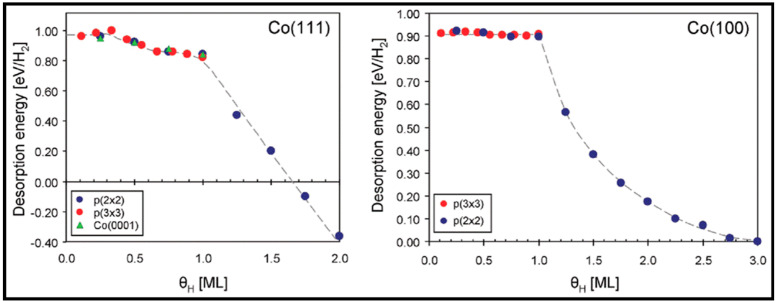
H_2_ desorption energies on the Co (111) and Co (100) surfaces, which are coverage-dependent [57].

The adsorption of carbon at various coverages on the Co (111) and Co (100) surfaces and their sub-layers was studied by Van Helden et al. [56]. No carbon migration to the subsurface layer occurs at low carbon coverage, but, upon increasing the coverage, the subsurface carbon geometries become more stable, and migration to the subsurface becomes more prominent, when higher carbon coverage causes strong lateral repulsions between the surface carbon atoms, which destabilizes the system. Although a low surface coverage of atomic carbon is expected under FT conditions, the formation of subsurface structures can enhance the electronic structure of the Co surface by influencing surface reactivity. Comparing the coverage on both Co (10$\bar{1}$0) and Co (11$\bar{2}$1) surfaces shows that although Co (10$\bar{1}$0) is less dense than the Co (11$\bar{2}$1) surface, less migration to the subsurface layer of Co (10$\bar{1}$0) occurs at high carbon coverage. In addition to the recent work, the Van Helden group also performed further calculations to understand the coverage dependence of hydrogen adsorption on the Co (11$\bar{2}$1) and Co (10$\bar{1}$0) surfaces [57]. In this work, they also investigated the role of defects on hydrogen adsorption by considering the Co (21$\bar{3}$1) and Co (22$\bar{4}$1) stepped surfaces. The resulting hydrogen adsorption energies are nearly the same at the Co (10$\bar{1}$0) and Co (11$\bar{2}$1) surfaces. Furthermore, the positive values indicate low stability compared to the gas phase hydrogen molecule, which implies the high mobility of the hydrogen atoms on these surfaces. According to the results in Figure 7, they concluded that hydrogen adsorption and desorption are coverage-dependent. They also compared hydrogen adsorption on the Co (11$\bar{2}$1) and Co (0001) surfaces and found that due to their similar structure, the adsorption energies on these two surfaces are the same, and hydrogen atoms are very mobile at low coverages. Moreover, the Co (21$\bar{3}$1) and Co (22$\bar{4}$1) surfaces were used to shed light on the key role of defects in the adsorption process. Since defects and step sites introduce pathways with much lower desorption activation energies, they can accelerate the adsorption of hydrogen. At higher hydrogen coverage, the lateral repulsions between hydrogen atoms lead to a further decrease in the adsorption energies. Hence, a broad range of adsorption sites with varying (mostly less stable) adsorption energies are available. It should be noted that, while the defect sites facilitate hydrogen adsorption, they cannot change the low coverage adsorption energies. Hydrogen (H_2_), as an important element in CO conversion, requires more detailed consideration, but it is clear that surface coverage has a remarkable impact on the binding energies of adsorbed species and reaction mechanisms. 

Recently, hydrogen adsorption, desorption, and dissociation on the Co (11$\bar{2}$0), (31$\bar{4}$1), (11$\bar{2}$1), and (10$\bar{1}$0) surfaces at distinct coverage were considered [58]. Lateral repulsive interactions were proposed to influence the adsorption structures, with increasing H_2_ coverage making their effect stronger. The reaction energies, activation barriers, and desorption energies of H_2_ dissociation on the above-mentioned surfaces are listed in Table 4. The H_2_ dissociation barriers are much smaller than their desorption energies for all coverages. Therefore, it can be concluded that H_2_ dissociation is preferable over desorption, independent of the H_2_ coverage, whereas the stability of hydrogen on the surface is affected by temperature and the H_2_ partial pressure. The results for the Co (31$\bar{4}$1) surface demonstrated the stabilization and increasing coverage through increasing the H_2_ partial pressure, whereas below 10 atm of H_2_ partial pressure, no H_2_ adsorption occurs on the Co (11$\bar{2}$1) surface. It is also described that, above room temperature, no H_2_ molecules adsorb at the surface. On the Co (11$\bar{2}$0) surface, adsorption occurs at low coverage, and the first H_2_ desorption starts below 10 atm of partial pressure, whereas, on the Co (10$\bar{1}$0), the most stability occurs at an H_2_ partial pressure of over 10 atm. Overall, increasing the H_2_ partial pressure as well as decreasing adsorption temperature stabilizes H_2_ adsorption and increases the surface coverage, particularly in the form of H atoms on the cobalt surfaces during the FT process. 

**Figure 8:**
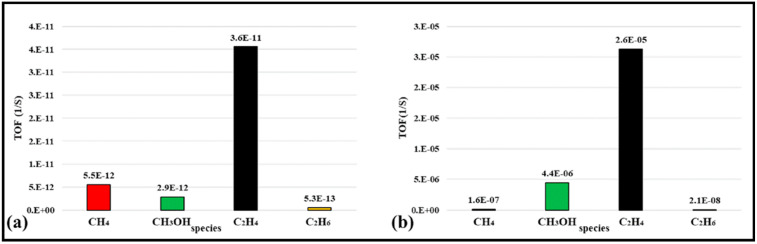
Turnover frequencies (TOF) for methane, methanol, ethylene, and ethane for the (**a**) non-coverage-dependent model and (**b**) coverage-dependent model. The studied reaction conditions are T = 500 K, PCO = 3.33 bar, and PH_2_ = 6.67 bar [31].

**Figure 9:**
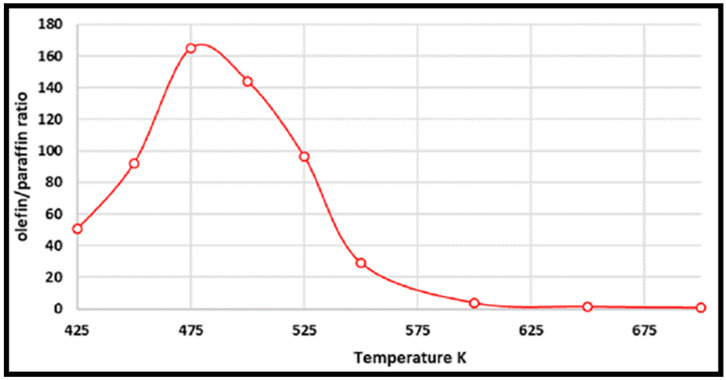
The olefin/paraffin ratio from temperature range of 425 to 700 K [31].

Coverage-dependent and independent models were used by Yao et al. to investigate the activity and selectivity of the Co (0001) catalyst for CO conversion in the FT process [31]. Both models demonstrated that the dominant mechanism on the flat Co (0001) surface is via CO insertion. The coverage-dependent model revealed that rapid direct CO dissociation is difficult on Co (0001) at high CH_x_ coverages. Hence, CH_x_ cannot occupy enough surface sites, which leads to a carbide mechanism. The calculated reaction pathways for CH formation in both the coverage and non-coverage models are the same. The main CH monomer formation pathway is through CO + H → CHO, CHO + H → CHOH, and CHOH → CH + OH. In the formation of C_2_ species, the dominant chain growth is related to the CO + CH_2_ coupling pathway, whereas the CH-CH interaction possibility is lower than the CH–CO interaction. The comparison of the selectivity between the coverage-independent model and the coverage-dependent model on the cobalt surface indicates high selectivity toward ethylene in both models (sketched in Figure 8). The selectivity follows the order of C_2_H_4_ > CH_4_ > CH_3_OH > C_2_H_6_ in the former model and nearly the same in the latter model (C_2_H_4_ > CH_3_OH > CH_4_ > C_2_H_6_). Thus, the formation of undesired methane product is rare, except in the presence of defect sites, which lead to a high selectivity toward methane. The surface coverage and product selectivity, as a result, are remarkably affected by temperature. As seen in Figure 9, the peak of the olefin/paraffin ratio is around the typically used temperature. At low temperatures, the surface activity reduces, and long-chain hydrocarbons and oxygenates are the main products. Additionally, high temperature also shows a negative influence, since increasing the temperature leads to a higher selectivity toward methane. 

**Figure 10:**
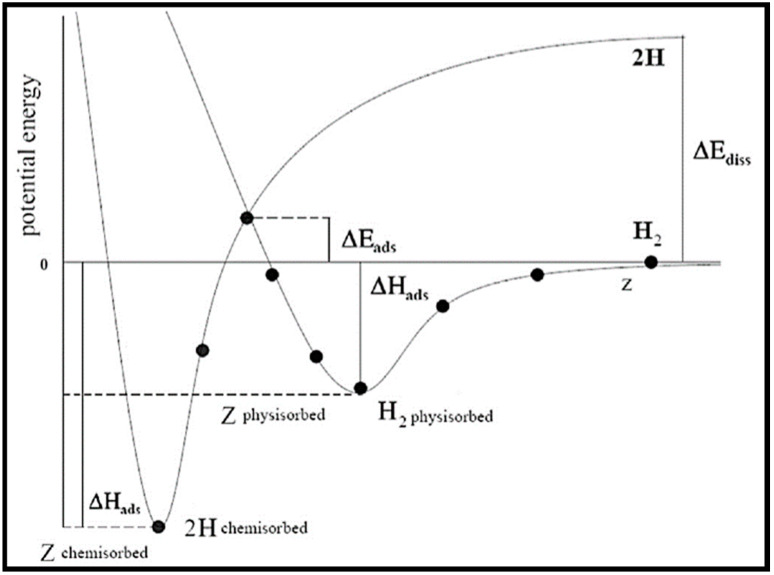
The hydrogen chemisorption (strongly bonded to the surface) and physisorption (weak interaction with the surface) paths on cobalt surface [59].

Hydrogen adsorption energies are calculated on three crystalline phases of metallic cobalt, namely, the (10$\bar{1}$0), (11$\bar{2}$0), and (11$\bar{2}$1) surfaces [59]. Surface free energies play a significant role in the adsorption states and, consequently, the adsorption energies in the order of Co (10$\bar{1}$0) > Co (11$\bar{2}$0) > Co (11$\bar{2}$1). The adsorption energies and distances from the surfaces for atomic and molecular hydrogen at different coverage states (0.25, 0.5, and 1 ML) are listed in Table 5. It is clear that, by increasing the surface coverage, the adsorption energies of both the hydrogen atom and molecule are slightly reduced. Figure 10 shows the physisorption of the hydrogen molecule (the weak interaction of molecular hydrogen with the surface) and the hydrogen atom chemisorption (the strong interaction and dissociation of hydrogen at the surface), in which the Z parameter is representative of the distance between hydrogen (atoms or molecules) and the cobalt surface. According to Table 5 and Figure 10, one can see that ZH_2_ is higher than ZH for all surfaces and all surface coverages. Additionally, DOS calculations reveal that the band gap narrows after both atomic and molecular adsorption. The Nakhaei Pour group also explored carbon monoxide adsorption energies on the three above-mentioned surfaces with distinct coverages [24]. They found the same order for the adsorption energies of carbon monoxide as that obtained for hydrogen (Co (10$\bar{1}$0) > Co (11$\bar{2}$0) > Co (11$\bar{2}$1)). The calculated adsorption energies [24] show that due to the increase in the repulsive interactions between CO molecules, the adsorption energies decrease with increasing coverage. 

As is known, FT synthesis starts with CO and H_2_ dissociative adsorption, followed by hydrogenation and the generation of CH_x_ (x = 1–3) intermediates and C + C coupling reactions. To explore the FT synthesis mechanism of CO conversion on the Co catalysts, the activation energies of C_1_ + C_1_ coupling and carbon hydrogenation on both flat and stepped Co (0001) surfaces were calculated [12]. Cheng et al. found that the adsorption of C_1_ species at stepped surfaces is favored in comparison to flat sites, and the transition states on the step sites are also more stable than those on flat surfaces. They also mentioned that while the barriers on flat surfaces are smaller, initial states and transition states are favored on step sites. The results for the reaction rates of the coupling pathways demonstrate that C + CH_3_ and CH_2_ + CH_2_ coupling are the most effective chain-growth pathways on step sites, while, on flat surfaces, CH + CH coupling is the fastest. Furthermore, the coupling of RC + C and RC + CH, in addition to the coupling of RCH + CH_2_ and RCH_2_ + C, also contributes to the chain growth. Recently, the chain growth and coupling reactions of C_1_ + C_1_ on Co (0001) were investigated by the Qi group [33]. The calculated adsorption energies and structural parameters displayed the top site and the hollow site as the most favorable adsorption site for CO and CH_x_, respectively. As is apparent from the main coupling reaction barriers reported in Table 6, the highest reaction barrier occurs in the C + C coupling reaction, while C + CH_2_ has the lowest barrier among C and CH_x_ (0–3) coupling reactions. Moreover, among all CH_x_ (0–3) + CH_x_ (0–3) coupling reactions, CH_2_ + CH_2_ has the lowest reaction barrier, and CH_3_ + CH_3_ has the highest reaction barrier. These results demonstrate the lower barriers of HCO insertion into CH_x_ than those of CO insertion, which at first glance implies its higher desirability. However, a volcano curve is observed between the activation barriers and adsorption energies in the central area of the table, which shows the lower activation energies. Further analysis by kinetic isotope effect coupled with theoretical calculations proposes that the CH_2_ + CO coupling reaction is a favorable chain propagation pathway, whereas CH_2_ + CO, HCO + CH, and CH + CH have relatively low barriers and high thermodynamic stabilities. An additional study on the increment of relative free energy by employing higher pressures indicates the stronger adsorption of CO and the larger site coverage of CO at a higher pressure, which, hence, induces more stable chain growth at higher pressure. 

The exploration of FTS mechanisms is important since CO conversion is an essential process, and adsorption of the surface species, CO activation, chain growth, and methanation are key processes. Many studies are devoted to these subtopics on cobalt-based catalysts, and Qi et al. summarized their obtained results as follows [6]: 

(i) The chemisorption energies of the most stable species on the flat and stepped Co (0001) as well as other facets of Co-based catalysts are listed in Table 7. The chemisorption energies are slightly different because of the different parameters employed in the evaluation, but they follow the same trend. As is apparent, the reported chemisorption energies are about 1 eV or more, which confirms the strong binding to the surfaces, except for CH_3_OH. Similarly, the adsorption energies on the stepped Co (0001) surface show that adsorbates more firmly bind to stepped surfaces than flat ones, except for CHO. Thus, this high binding lowers the activation barrier of the bond-breaking steps and leads to more active CO activation [60]. However, pre-adsorbed oxygen (1/4 ML) lowers the adsorption energy of CO on stepped surfaces [61]. The CO coverage can influence the chemisorption energies of other species on the surfaces, and various CO coverages of 1/4, 1/3, and 1/2 ML on the Co (0001) are summarized in Table 7 (left). The energies on the 1/4, 1/3, and 1/2 ML pre-covered surfaces from [31,62,63] are displayed in parentheses. It is evident that the adsorption energies on pre-covered surfaces are smaller than those on clean surfaces. The stability of molecules is reduced through the repulsive interaction among the adsorbates. In the case of COH, the reported chemisorption energy by the Helmen group indicated a contrasting result. They attributed these unexpected results to the interaction of the O atom in the pre-covered CO with the O–H of COH, the interaction of which stabilizes the hydrogen bond and enhances the chemisorption energy [64]. Table 7 shows the adsorption energies on several facets of the Co catalyst from [21,65,66]. Comparing C, O, and CO adsorption on the three facets of the Co ($11\bar{2}0$), Co ($11\bar{2}4$), and Co ($10\bar{1}2$) surfaces considered in [66] reveals that unlike CO adsorption energy, which does not notably vary on different surfaces, O and C adsorption energies can significantly change. In addition, binding oxygen to the surface slightly alters the Co structure due to Pauli’s repulsion to oxygen. Generally, the adsorbates more strongly bind to the surface of Co ($10\bar{1}2$) due to lower coordination numbers and the presence of favorable fourfold hollow sites [21]. It is also claimed that the relation of binding strength to structure sensitivity is more noticeable for less saturated adsorbates. Hydrogen adsorption does not indicate strong dependence on Co surface structures, even though it has a higher value on the Co ($11\bar{2}0$) and Co ($10\bar{1}2$) facets, as reported by Li and co-authors [21]. It is deduced that, unlike the low influence of surface structure on the chemisorption, CO coverage causes a notable reduction in adsorption energy.

(ii) Table 8 reports several calculations of CO activation, the first step in FTS, through different mechanisms. It is obvious that direct CO dissociation on the flat Co (0001) is difficult, whereas active sites on stepped surfaces are desirable for direct CO dissociation. The CO dissociation barrier on the double-stepped Co (0001) surface studied by Hu and co-workers is lower than that on the flat Co (0001) [67], owing to the interaction of CO with the surface via both C and O atoms, leading to stronger C–O bond activation. It is noteworthy that the carbide mechanism on the double-stepped Co (0001) surface significantly contributes to the formation of C_1_ species. Moreover, the calculated CO dissociation barrier reduces in the order of Co ($11\bar{2}0$) > Co ($10\bar{1}2$) > Co ($11\bar{2}4$), since C and O atoms more strongly bind to defect sites, and, hence, CO dissociation is facilitated [66]. Furthermore, the Shetty group proposed a six-fold (F6) novel site for CO dissociation on the Co ($10\bar{1}0$) surface, with a remarkably lower activation energy than on the flat Co (0001) surface [68]. Therefore, direct CO dissociation is structure-sensitive, and active sites are needed for direct CO dissociation to occur. The preferable route of CO dissociation goes through HCO, and HCO* + H* → CH_2_O* + * is the rate-determining reaction step in the mechanism. Inderwildi et al. found a similar pathway via HCO, with a hydrogenation to CH_2_O and following C–O bond cleavage of CH_2_O that only require a very low activation barrier to be overcome [69]. The C–O bond cleavage in HCO is also facilitated relative to the higher barrier of direct CO dissociation, whereas Saeys and co-workers reported similar results for C–O bond cleavage [70]. Thus, CO hydrogenation to CHO and CH_2_O weakens the C–O bond and lowers the required activation barriers for its cleavage. Hence, it can be inferred that the hydrogen-assisted mechanism governs the CO activation process on flat Co (0001). Furthermore, different activation barriers through the CHO route on the Co (0001), Co ($10\bar{1}2$), and Co ($11\bar{2}0$) surfaces showed the structure sensitivity of hydrogen-assisted CO dissociation [21]. However, the overall barrier of the H-assisted route via HCO on the Co (21$\bar{3}$1) is slightly lower than that via direct dissociation [71], and the overall barrier of both H-assisted and direct dissociation on Co ($10\bar{1}2$) and Co ($11\bar{2}0$) is almost the same [16,21]. 

**Table 7:** Chemisorption energies of various intermediates relevant in FTS on different facets of cobalt catalysts [6].

Species	E (eV) on Flat Surface	E (eV) on Stepped Surface	$\mathrm{Co}\;(11\bar{2}0)$	$\mathrm{Co}\;(11\bar{2}4)$ ^a^	$\mathrm{Co}\;(10\bar{1}1)$ ^b^	$\mathrm{Co}\;(10\bar{1}2)$	$\mathrm{Co}\;(10\bar{1}0)$ ^b^	$\mathrm{Co}\;(11\bar{2}1)$ ^b^	Co (111) ^b^	Co (100) ^b^	Co (311) ^b^	Co (110) ^b^
C	6.92 ^c^, 6.71(5.61) ^d^, 6.83 ^e^, 6.46 ^f^, 6.62 ^g^, 6.54 ^h^	7.44 ^f^, 7.53 ^g^, 7.32 ^h^	7.09 ^a^, 7.22 ^b,e^	7.43	8.15	7.81 ^a^, 7.85 ^b,e^	7.07	7.55	6.80	8.01	7.69	7.25
O	5.97 ^c^, 5.89(5.72) ^i^, 5.43(4.34) ^d^, 5.65 ^e^, 5.34 ^f^	5.59 ^f^	5.44 ^a^, 5.63 ^b,e^	5.79	6.06	5.97 ^a^, 6.04 ^b,e^	5.70	5.85	5.61	5.99	5.74	5.50
H	2.90 ^c^, 2.85(2.61) ^i^, 2.88(2.29) ^d^, 2.78 ^e^, 2.72 ^f^, 2.94 ^g^, 2.85(2.60) ^j^	2.74 ^f^, 2.90 ^g^	2.66 ^e^	-	0.58	0.47 ^b^, 2.73 ^e^	-	0.52	-	0.44	0.49	0.43
CO	1.81 ^c^, 1.72(1.68) ^i^, 1.88(0.78) ^d^, 1.64 ^e^, 1.66 ^f^, 1.66 ^a^, 1.77 ^k^, 1.70(1.67) ^j^	1.90 ^f^, 1.42 ^k^	1.65 ^a^, 1.65 ^b,e^	1.71	1.85	1.70 ^a^, 1.77 ^b,e^	1.70	1.82	1.61	1.71	1.71	1.61
CH	6.46 ^c^, 6.43(6.22)9, 6.31(5.48) ^d^, 6.30 ^e^, 5.99 ^g^, 6.54 ^h^, 6.72(6.51) ^j^	6.33 ^g^, 6.88 ^h^	6.47 ^e^	-	7.02	6.84 ^b,e^	-	6.37	-	4.68	6.56	6.37
HCO	2.22 ^c^, 2.24(0.37) ^d^, 2.14 ^e^, 2.20 ^a^, 2.17(1.93) ^j^	2.82 ^l^	2.56 ^e^	-	2.67	2.97 ^b,e^	-	2.40	-	2.80	2.68	2.54

a [66], b [65], c [70], d [72], e [21], f [73], g [74], h [12], i [63], j [64], k [61], l [60].

**Table 8:** The CO activation energies of elementary steps on various cobalt-based catalysts [6].

No.	Elementary Steps	Co (0001)	Stepped Co (0001)	Other
1	CO → C + O	3.80 ^a^, 2.28 ^c^, 2.82 ^e^, 2.79 (4.11) ^g^, 3.37 ^m^ 2.40 ^h^, 2.70 ^i^, 2.46 ^k^	1.20 ^d^, 1.61 ^i^	0.70 ($10\bar{1}0$) ^b^, 2.02 ($11\bar{2}0$), 1.27 ($10\bar{1}2$), 0.92 ($11\bar{2}4$) ^h^1.34 ($10\bar{1}2$) ^k^, 1.39 ($11\bar{2}0$) ^k^, 1.07 ($11\bar{2}1$) ^l^, 1.21 ($10\bar{1}1$) ^l^, 1.79 ($10\bar{1}0$) ^l^, 2.48 (111) ^l^, 1.56 (311) ^l^, 1.47 (110) ^i^
2	CO + H → COH	1.30 ^a^, 1.80 ^f^, 1.55 ^m^	2.29 ^d^, 1.46 ^f^	
3	COH → C + OH	3.26 ^a^, 2.68 ^m^		
4	COH + H → HCOH	0.46 ^a^, 0.85 ^f^	0.77 ^f^	
5	CO + H → HCO	1.43 ^a^, 1.51 ^c^, 1.31 ^e^, 1.31 ^f^, 1.18 ^k^, 1.25 ^m^	0.09 ^d^, 0.77 ^f^	0.61 ($10\bar{1}0$) ^b^, 0.95 ($11\bar{2}0$) ^k^, 1.13 ($10\bar{1}2$) ^k^, l,0.59 ($10\bar{1}1$) ^l^, 0.63 ($11\bar{2}1$) ^l^, 0.76 (311) ^l^, 0.71 (110) ^l^, 1.07 (100) ^i^
6	HCO → CH + O	0.95 ^a^, 0.93 ^c^, 1.00 ^e^, 0.73 ^k^, 0.90 ^m^	1.36 ^d^	0.52 ($10\bar{1}0$) ^b^, 0.72 ($11\bar{2}0$) ^k^, 1.04 ($10\bar{1}2$) ^k,l^, 0.59 ($10\bar{1}2$) ^l^, 0.63 ($11\bar{2}1$) ^l^, 0.76 (311) ^l^, 0.71 (110) ^l^, 1.07 (100) ^l^
7	HCO + H → HCOH	0.93 ^a^, 1.23 ^f^, 0.80 ^m^	1.59 ^f^	
8	HCOH → CH + OH	1.10 ^a^, 0.73 ^m^		
9	HCOH + H → CH_2_OH	0.82 ^f^, 0.71 ^m^	0.43 ^f^	
10	HCO + H → CH_2_O	0.15 ^a^, 0.62 ^c^, 0.45 ^e^, 0.55 ^f^, 0.24 ^m^	0.61 ^d^, 0.71 ^f^	
11	CH_2_O → CH_2_ + O	1.63 ^a^, 0.70 ^c^, 0.85 ^e^, 0.95 ^f^, 0.95 ^m^	1.22 ^d^, 0.85 ^f^	
12	CH_2_O + H → CH_2_OH	1.27 ^f^, 1.20 ^m^	1.34 ^f^	
13	CH_2_OH → CH_2_ + OH	0.83 ^m^		
14	CH_2_OH + H → CH_3_OH	0.98 ^f^	0.82 ^f^	
15	CH_2_O + H → CH_3_O	0.86 ^f^	0.45 ^f^	
16	CH_3_O + H → CH_3_OH	1.45 ^f^	1.24 ^f^	
17	CH_3_OH → CH_3_ + OH	1.47 ^f^	1.07 ^f^	
18	CH_3_CHO → CH_3_CH + O	0.52 ^c^, 0.63 (0.73) ^j^		

^a^ [72], where the data in the parentheses were calculated on the surface with 0.5 ML pre-covered CO. ^b^ [75]. ^c^ [70]. ^d^ [67]. ^e^ [69]. ^f^ [60]. ^g^ [61], where the data in the parentheses were calculated on O pre-covered surface. ^h^ [66]. ^i^ [76]. ^j^ [63], where the data in the parentheses were calculated on Co (0001) with 1/3 ML CO coverage. ^k^ [21]. ^l^ [65]. ^m^ [64], where the surface with 1/4 ML pre-covered CO.

(iii) FTS includes a large number of intermediates and, according to the main chain propagation mechanism, different products are formed. Several efforts were devoted to the formation of chains containing C_2_ and C_3_ species [52,65,67,69,72,77,78]. All possible C_1_ + C_1_ coupling reaction pathways were studied by Hu and co-workers for the pathways in the carbide mechanism [12]. The left panel of Table 9 shows that all the C_1_ + C_1_ coupling pathways on the flat surface have smaller barriers than those on the stepped surface, except for CH_2_ + CH_2_ and CH_2_ + CH_3_. Considering the differences in the adsorption energies of the adsorbed reactants indicates that the C + C coupling reaction occurs on the step sites rather than the flat surface and the two major chain growth pathways at the step sites belong to CH_3_ + C and CH_2_ + CH_2_. Shortly after, Hu et al. investigated C_2_ hydrogenation and C_2_ + C_1_ and C_3_ + C_1_ coupling reactions on the stepped Co (0001) [79]. They indicated that two RC + C and RC + CH reactions may play a role in the chain growth, whereas the coupling reactions RCH_2_ + C and RCH + CH_2_ are the most likely chain-growth pathways for C_2_ + C_1_, especially for chain lengths *n* ≥ 2. It was also shown that C–C coupling reaction barriers do not alter for chain lengths larger than 2. The CO insertion mechanism on cobalt-based catalysts was also studied by Saeys and co-workers [70]. The right panel of Table 9 lists the calculated barriers of the elementary steps for CO insertion. As can be seen, the barrier of CO insertion into CH_2_ and CH is comparable to the C_1_ + C_1_ coupling in the carbide mechanism. In addition, it is revealed that at higher CO coverages, the activation barrier of CH_2_ + CO coupling decreases, in contrast to the increasing value of the C–O scission barrier [63]. Hence, it can be deduced that the insertion mechanism is facile on Co-based catalysts at high CO coverage. 

(iv) It is vitally important to suppress methane formation during CO conversion on cobalt-based catalysts. A number of theoretical works on CH_4_ formation and the activation energies for CH_x_ hydrogenation are summarized in Table 10. The controlling step in CH_x_ (x = 0–3) hydrogenation on both flat and stepped Co (0001) surfaces is CH_3_ hydrogenation [74]. Very low barriers are needed to be overcome to decompose CH_2_ to CH or hydrogenate it to CH_3_, and CH_2_ is, hence, the least stable CH_x_ intermediate, which was also confirmed by Hu et al. [12]. Overall, although CH_3_ hydrogenation has a high barrier, the sequential hydrogenation of carbon to CH_4_ can readily occur. Furthermore, CH_x_ (x = 0–3) hydrogenations on Co (0001), Co ($10\bar{1}2$), and Co ($11\bar{2}0$) surfaces are not notably sensitive to the structure, as CO activation is not significantly structure-sensitive [21]. The exploration of all possible reaction routes by Qi et al. suggested that CO hydrogenation to HCOH followed by HCOH dissociation forms CH, while CH_4_ was formed by further hydrogenations [64]. 

**Figure 11:**
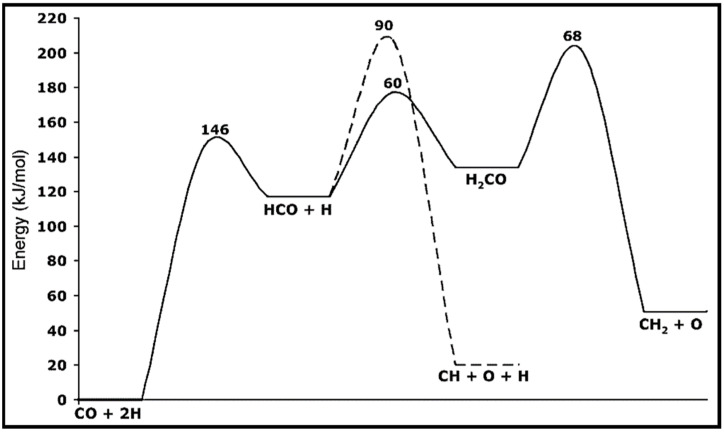
Energy profile related to the hydrogen-assisted CO activation route on Co (0001) surface [64].

**Figure 12:**
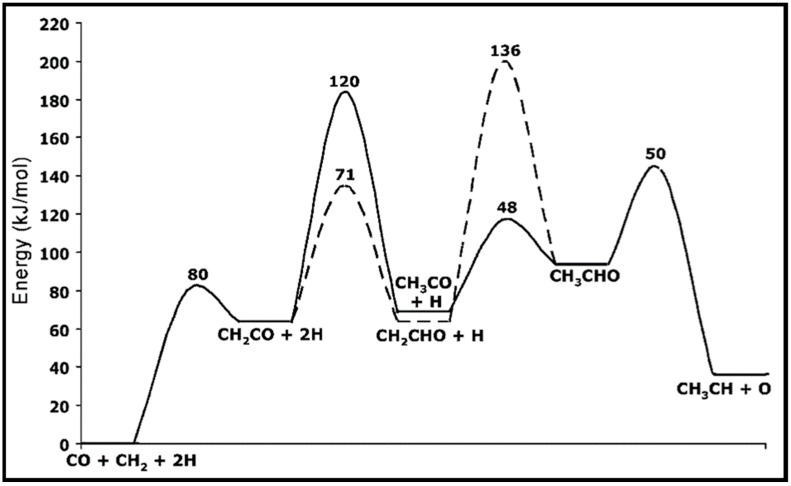
Energy profiles of the propagation cycles related to proposed propagation cycle for the CO insertion mechanism [64].

The calculations of CO activation on the Co (0001) surface indicated low concentrations of surface C or CH_2_ species and high energy barriers to CO hydrogenation. Hence, Zhuo et al. suggested a propagation cycle that starts with CO insertion into surface RCH groups [70]. Since the C–O bond scission is the key step, the influence of CO hydrogenation and its coupling with CH_x_ on the C–O dissociation barrier is considered. They illustrated that hydrogenation has a higher barrier than CO dissociation. As can be seen from the energy profile sketched in Figure 11, the formyl intermediate (HCO) is relatively unstable, and hydrogenation lowers the C–O dissociation barrier. Further investigations suggested the kinetic possibility of CO insertion into RCH at low coverage. Due to the strong interaction of the CH_2_ group with the Co (0001) surface, a dissociation mechanism over a Co atom is not more favorable, whereas CO insertion into CH_2_ and the formation of CH_2_CO are highly desirable. Two pathways are reported for the conversion of CH_2_CO to CH_3_CHO, via CH_3_CO and CH_2_CHO, which compete together. The energy profile of the propagation cycle is presented in Figure 12. Due to the easy C–O dissociation on the surface, the aldehyde hydrogenation to produce alcohol seems difficult, while the hydrogenation or dehydrogenation of the RCH intermediates looks feasible. Moreover, the coupling of CO with the RCH group at the surfaces became faster, even for low RCH coverages.

**Figure 13:**
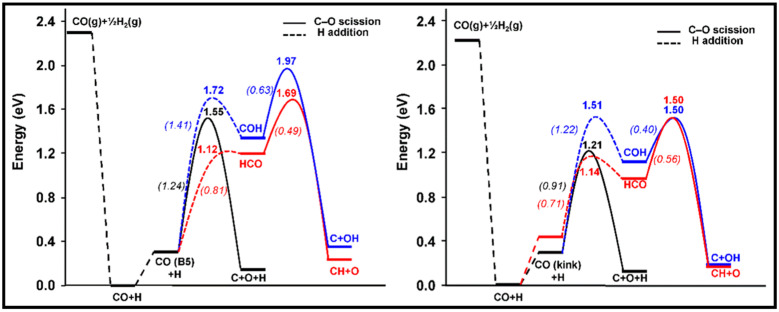
Potential energy profile for direct and H-assisted CO dissociation on the (**left** panel) the B5-B site of Co (221) and (**right** panel) the kink site of Co (321). The zero-energy reference belongs to adsorbed CO and H, in their respective lowest energy sites on studied surface [20].

**Figure 14:**
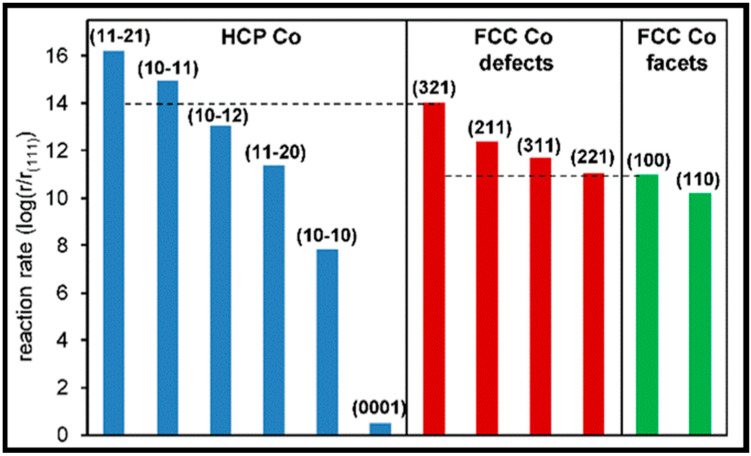
The CO dissociation rates for low coverage at 500 K on three HCP Co facets, FCC Co facets, and FCC Co defect sites. The results are normalized to the CO dissociation rate calculated for FCC Co (111) [20].

In a recent study, Petersen and co-workers studied the direct and H-assisted dissociation of CO at the kink and step sites on FCC Co (321) and Co (221). The potential energy profiles for the dissociation reactions on Co (221) and Co (321) and their corresponding activation energies are reported in Figure 13 and Table 11, respectively. The H-assisted pathways, via HCO or COH intermediates, are not energetically favorable in comparison to direct dissociation, which is the preferred mechanism of CO activation on these surfaces. The activation energy for breaking the C–O bond at the kink site (Co (321) surface) is the same for both H-assisted pathways, but it is still higher than for direct CO dissociation. Petersen et al. also emphasized that the steady-state coverages of adsorbed CO and H and the availability of free active sites significantly influence the dominant mechanism. The comparison among the reaction rates of several Co surfaces (HCP Co, FCC Co defects, and FCC Co facets) represented in Figure 14 confirmed that the step and kink defect sites are more active than other facets in direct CO dissociation at low coverage, and the preferred mechanism for CO activation on FCC and HCP Co is direct CO dissociation. 

**Figure 15:**
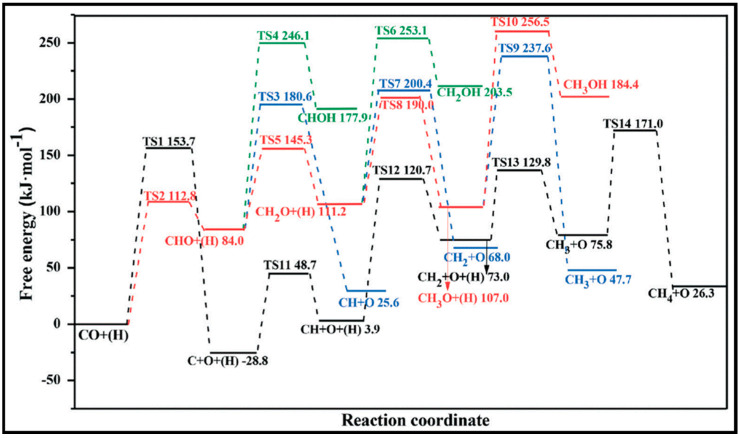
The free energy profile for CO activation through direct and H-assisted dissociation and formation of CH_4_ and CH_3_OH formation at 500K [81].

Hexagonal close-packed (HCP) Co exhibits higher activity in the FTS process. Since the open Co ($10\bar{1}1$) surface contains 35% of the total surface area of the exposed HCP, it may be suitable to be employed in CO conversion. Hence, the preferred mechanism of the C–C chain growth as well as the CH_4_ and C_2_H_4_ hydrocarbon selectivity was considered on the Co ($10\bar{1}1$) surface by Liu et al. [81]. They illustrated that the favorable route to form CH goes through direct CO dissociation, rather than the H-assisted CO route, and the CHO intermediate is not stable at low coverage. As can be seen in Figure 15, CHO dissociation into CH + O and CHO insertion into CH compete with each other and are more favorable than CH_3_OH formation. It can also be deduced that CH is the favored monomer among the CH_x_ (x = 1–3) species, since CH formation has a low activation free energy. In addition, CH and CH_2_ self-coupling are parallel and desirable reactions to produce C_2_H_2_ and C_2_H_4_, respectively. CH self-coupling and CH_2_ self-coupling via the carbide mechanism mainly contribute to ethane formation. Three successive hydrogenations from CH form CH_4_, and the CH + H→CH_2_ coupling reaction is the rate-determining step. 

### 2.2. Cobalt-Promoted Catalysts

Theoretical studies on cobalt-promoted catalysts (e.g., Mn) indicated that promotion has a stabilizing effect on the adsorption of CO, C, H, O, and CH_x_, whereas it also reduces the CO dissociation barrier, which makes higher intrinsic activity [30].

Alumina-supported cobalt catalysts and Mn-promoted Co catalysts were recently examined and compared with cobalt-based materials. Nguyen et al. adsorbed the CO molecule on a Co_4_ cluster and the Co_4_/Al_2_O_3_ system [82]. Their results showed that the adsorption energy on Co_4_/Al_2_O_3_ is lower than the dissociation energy of the C–O bond, whereas the adsorption of CO on Co_4_/Al_2_O_3_ also alters the pathway to the available final products during hydrogenation. Yang et al. studied different metal promoters, including Rh, Ir, Ga and Sb, to modify the Co/Al_2_O_3_ system and evaluate their effects on FTS reactions. Their work included kinetic analyses, and the results showed that promoters mainly affect the surface CH_x_ concentration to alter the CO reaction rate, whereas the chain-growth rate constants affect the product distribution. This kinetical study reveals that Ir- and Rh-promoted catalysts have larger numbers of active sites, but the Sb- and Ga-promoted catalysts have fewer active sites, which is consistent with the dispersion data. In addition, the quantities of CH_x_ intermediates showed a similar trend with that of surface CO over these catalysts [83]. Shortly after, Pedersen and co-workers considered Mn-promoted Co catalysts for CO hydrogenation [30]. They confirmed that the presence of Mn has a remarkable effect on increasing the adsorption energies of all the investigated species, except CH_4_ (see Table 12). Both Mn-terminated (Mn/Co) surfaces and non-direct Mn-adsorbate Co/Mn/Co surfaces display high adsorption energies, although the former surfaces achieve a stronger effect. According to experimental studies, Mn promotion caused an increase in CO binding to the surfaces [84]. For such systems, the carbide mechanism is still appropriate, which is used for activation and methane formation. Daga and co-workers studied the effects of sulfur-covered cobalt surfaces on the selectivity of FTS [85], indicating that sulfur has an effect on the Co (111) selectivity and shows a comparable increase in adsorption energies. The adsorption energies for CO and the intermediate species of methane production were studied on Co (001) by Torkashvand et al. [50], whereas Vasqueze-Parga et al. studied the adsorption energies for CO on transition metal surfaces [86] and calculated that CO adsorbed on hcp (0001), (10$\bar{1}$0), and (11$\bar{2}$0) Co surfaces with energies of −1.48, −1.53, and −1.58, respectively. 

**Figure 16:**
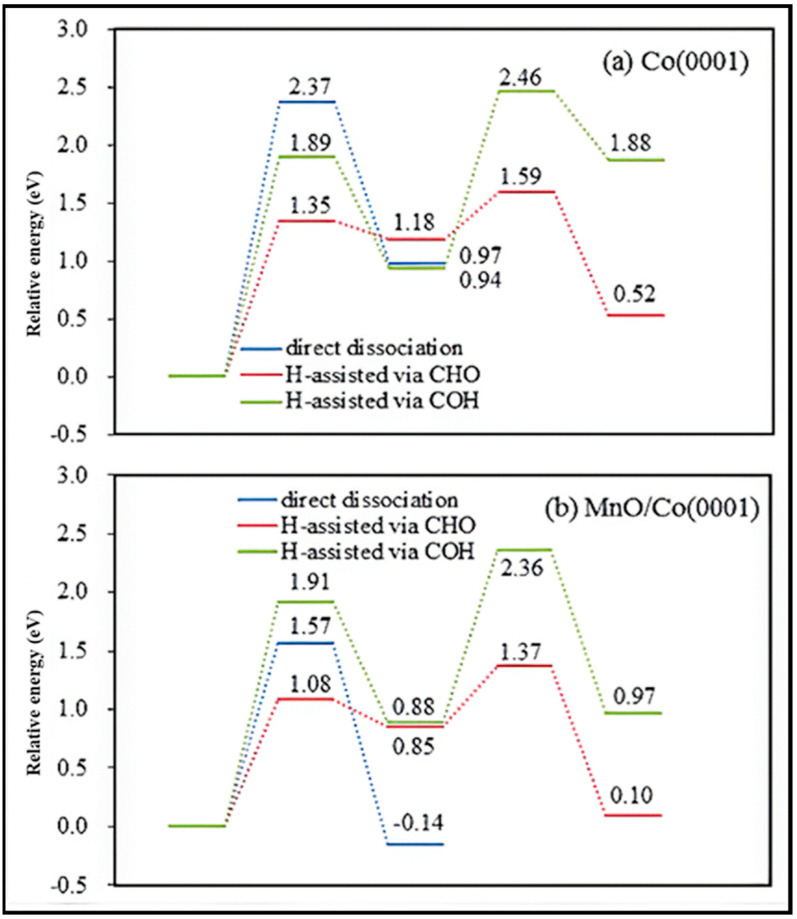
Potential energy profiles for CO dissociation through two H-assisted and direct paths on Co (0001) and MnO/Co (0001) [19].

**Figure 17:**
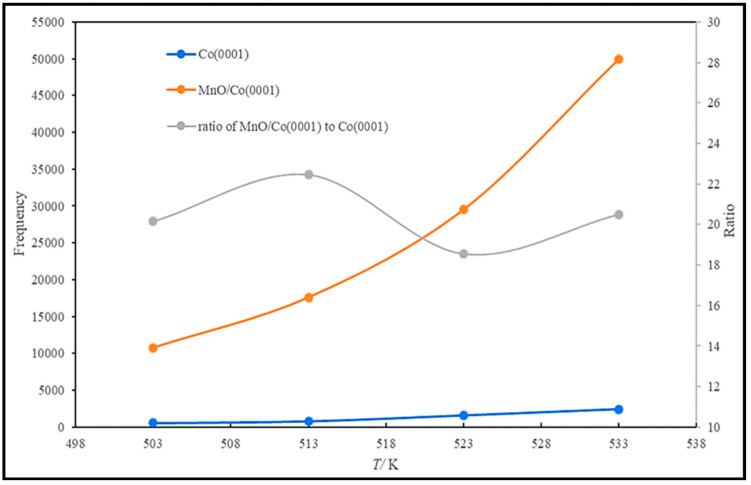
The total frequencies of C–O bond fracture as a function of temperature on Co (0001) and MnO/Co (0001). Data are collected in 1.2 s of time [19].

CO activation and oxygen removal on the Co (0001) and Mn-promoted Co (0001) surfaces were compared in detail [19]. The presence of MnO increases the stability of the transition state and decreases the reaction barrier, which improves the efficiency of dissociation. The adsorption energy calculation shows that the adsorption of CO, C, O, CHO, H_2_O, and CO_2_ is enhanced on MnO/Co (0001), in contrast to the weakened H and CH adsorption. No obvious effect on COH and OH adsorption was observed on the MnO/Co (0001) surface, and the partial density of states of the d band (d-PDOS) of Co on this surface reveals that the Mn addition causes the d-band center of Co atoms to shift away from the Fermi level. Hence, the bond strength between the Co and adsorbate weakens, and, consequently, the adsorption strength of H and C, which are not directly bonded to Mn atoms, reduces. It is also indicated that H* is the main form of H_2_, since hydrogen molecules can be easily activated and dissociated into adsorbed H (H*) on both surfaces. The potential energy profiles of CO activation on Co (0001) and MnO/Co (0001) via three dissociation paths are plotted in Figure 16. On the Co (0001) surface, CO is more likely to hydrogenate to form CHO than COH, and the C–O bond fracture of CHO is easier than that of COH, while direct dissociation is generally difficult. It is clear that the energy barrier of CO hydrogenation to CHO is reduced by the addition of MnO, even though the energy barrier of hydrogenation to COH is not affected. Similar to the Co (0001) surface, the fracture of the C–O bond of CHO on MnO/Co (0001) is more facile than that of COH. Therefore, it can be inferred that H-assisted CO dissociation takes place on both surfaces, but direct dissociation tends to occur on MnO/Co (0001). More investigation clarified that the preferred activated path of CO goes through the CHO intermediate at all studied temperatures. However, the percentages of CO direct dissociation slightly increase, from 0.0074 at 503 K to 0.0134 at 533 K, when the temperature is increased. Furthermore, Mn causes inhibition of the reverse reaction of the COH intermediate and, as a result, improves the efficiency of CO dissociation. When we consider the frequency of C–O bond scission as a function of temperature, as plotted in Figure 17, it is clear that the bond fracture is improved by about 20 times through the addition of the Mn promoter. 

**Figure 18:**
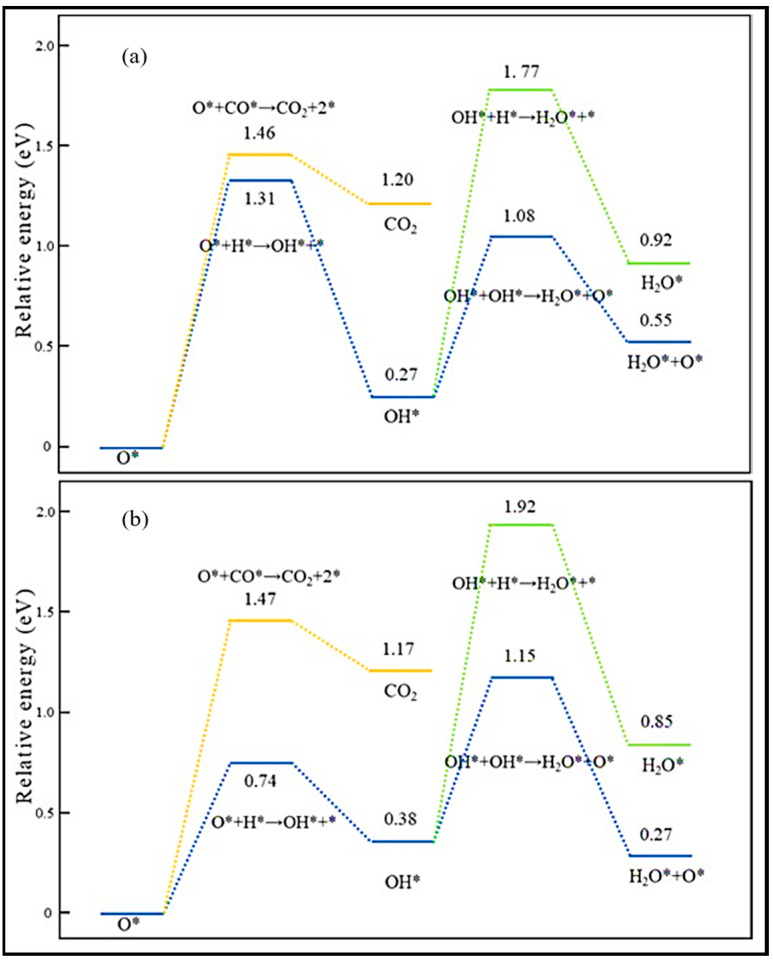
The potential energy diagram for possible O removal pathways on (**a**) Co (0001) and (**b**) MnO/Co (0001) [19].

As is known, oxygen is removed from the surface in the form of H_2_O or CO_2_. In the case of H_2_O, the two possible pathways include the gradual hydrogenation of O and the disproportionation of OH to produce H_2_O. Figure 18 describes the potential energy profiles of three oxygen removal routes: the adsorbed O gradually hydrogenates to H_2_O, OH disproportionates to H_2_O, and CO oxidizes to CO_2_ on both surfaces. The adsorbed O* removal tends to proceed through the formation of H_2_O via the OH disproportionation on both Co (0001) and MnO/Co (0001). The activation energy of OH disproportionation drops with the addition of MnO. Owing to the fact that H_2_O is more stable on MnO/Co (0001), OH disproportionation is exothermic on MnO/Co (0001) but endothermic on Co (0001). Also, the low WGS activity of the Co catalyst does not vary with Mn addition to the catalyst. Further calculation of the CO_2_–H_2_O ratio displayed in Table 13 unveils that CO_2_ formation is rising with temperature on Co (0001), but O removal mainly occurs via H_2_O at all temperature points on MnO/Co (0001). So Mn addition leads to lowering CO_2_ formation and rising C element selectivity, which is the advantage of the Mn promoter. 

### 2.3. Cobalt Carbide 

It was suggested that various carbon products such as Co_2_C form during the FTS process, which reduces CO conversion [87]. Hence, it is important to identify the nature of resilient carbon species under FTS conditions. Tan et al. both experimentally and theoretically investigated the stability of different types of deposited carbon on the Co catalyst [8]. Their calculations indicated that CO conversion increases during the first 24 h of FTS and gradually decreases after 200 h due to catalyst deactivation. Furthermore, both theoretical and experimental results showed the formation of graphene islands and a p_4_g surface carbide under FTS conditions, which are thermodynamically stable. It should be mentioned that, from the obtained binding energy values (Table 14), large graphene islands are the most stable form. Additionally, a p_4_g surface carbide forms as the near-edge hcp hollow site carbon sinks into the Co surface, which is also highly favorable. The interaction of carbon with Co negatively affects the activity of the catalysts in the FT process. Therefore, tailoring the stability of carbon on the cobalt surfaces is of crucial importance. In this regard, Valero and Raybaud investigated carbon deposition on Co (11$\bar{2}$1), Co (22$\bar{4}$1), and Co (11$\bar{2}$2) surfaces [88]. They found strong reconstruction associated with the insertion of C in the Co (11$\bar{2}$1) surface at a low Anderson−Schulz−Flory (ASF) coefficient, while, at a high ASF coefficient, the oligomeric C species covers the Co (11$\bar{2}$1) surface as long-chain alkanes or graphene precursors. The carbon deposition energies on the stepped Co (11$\bar{2}$2) and Co (22$\bar{4}$1) surfaces and the Co (11$\bar{2}$1) terrace are reported in Table 15. Owing to the triangular rearrangement of Co (22$\bar{4}$1), similar to that of close-packed Co (11$\bar{2}$1), this surface shows the most exothermic deposition energies. As can be seen, the adsorption on stepped Co (22$\bar{4}$1) and Co (11$\bar{2}$2) surfaces is larger in the case of low carbon coverages (one or two C atoms per unit cell), while the gap between the flat and stepped surfaces tends to decrease for higher carbon coverages (three and four C atoms per unit cell). It is believed that carbon coverage alters the nature of active sites on the Co catalyst, where the slow deactivation of Co-based catalysts occurs because of the formation of a graphene overlayer. Carbon deposition occurs on both terraces and steps, which rapidly prevents CO dissociation. 

As stated before, tailoring the carbide phase formation and its effect on the products during Fischer–Tropsch (FT) synthesis is important, since Co-carbide is a possible cause of deactivation [89,90]. In work by Hu et al., the activity and selectivity of Co-carbide are investigated in comparison to those of the Co catalyst [9]. They reported that the CO dissociation barrier on the carbide surface is higher than on the metal surface, which suggests the lower activity of the Co-carbide surface due to the reduction in the binding strength of the C and O atoms on the surface. Furthermore, the transition from metallic Co to carbide phases (Co_2_C or Co_3_C) leads to increased methanation at higher temperatures. Thus, the Co-carbide surface performance is worse than the performance of metallic Co catalyst surface, although it has a higher CH_4_ selectivity. However, the transition state (TS) structures of the C_1_ + C_1_ coupling and C_1_ hydrogenation on the Co-carbide surfaces are the same as those on the stepped Co surface. 

**Figure 19:**
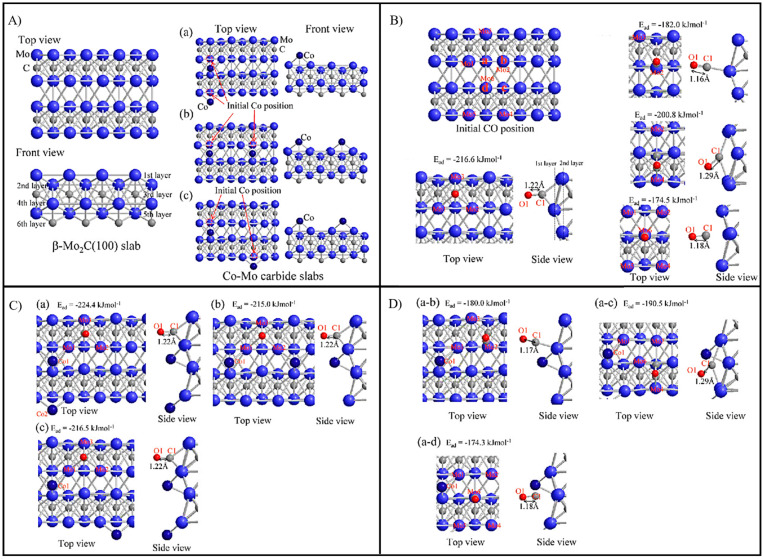
(**A**) The optimized structures of the Mo_2_C (100) slab from top and front views. The position of Co atoms is indicated by dashed circles. (**B**) Adsorption structures of CO on the -Mo_2_C (100) slab from top and side views before optimization. (**C**) Top and side views of the adsorption structures of CO on the Mo_2_C (100) carbide. (**D**) Top and side views of the adsorption sites [27].

The CO hydrogenation on the β-Mo_2_C (100) and Co-Mo carbide slabs was studied by Tominaga and co-workers [27]. The surface structures and adsorption sites on the surfaces are depicted in Figure 19a and Figure 19b,c, respectively. The results show that CO adsorbs on all the presented sites of β-Mo_2_C (100), and H_2_ adsorbs on the surface in competition with CO. The Co-Mo carbide is different, which facilitates H_2_ dissociation with less CO poisoning than the β-Mo_2_C (100) slab. Both hydrogen addition and C–O bond cleavage, two steps in hydrogenation, were investigated on both surfaces. On the Co-Mo carbide surface, hydrogen bonds to the carbon of CH_2_O and enhances hydrogenation to directly form CH_3_, while hydrogen does not bind to the carbon atom of CH_2_O for CH_2_ formation. In addition, C–O bond cleavage occurs during hydrogenation on both surfaces. CO hydrogenation is energetically favorable on the Co-Mo carbide surface. Moreover, according to electronic structure considerations, the addition of Co improves CO activation because of an increasing number of electrons.

In the CO hydrogenation under the FTS process, cobalt carbide (Co_2_C) coexists with Co metal [91,92]. The presence of the Co_2_C catalyst is responsible for CO associative adsorption, while Co metal causes CO dissociative adsorption and alkyl formation [78,93,94,95]. As the activity and selectivity of cobalt carbide-based catalysts in the conversion of syngas are promising, Chen et al. studied the activity of different Co_2_C surfaces on CO direct dissociation [3]. They considered several orientations of Co_2_C surfaces, including the (001), (100), (010), (101), (011), (110), and (111) surfaces. They found that CO dissociates through the direct (CO → C + O) or H-assisted CO route (CO + H → HCO → CH + O) but that H-assisted dissociation does have a significant role on the cobalt carbide surface. In the direct dissociation of CO on the Co_2_C surfaces, the reaction energies range from exothermic to endothermic owing to the significant sensitivity to the structure (Table 16). They also examined CO activation on defective (carbon vacancies) Co_2_C surfaces, which act as a promoter for direct CO dissociation. The results showed that (110)-C_1_A and (110)-C_2_B structures have the lowest CO dissociation barriers and, consequently, high activity. However, the low concentration of carbon vacancies, owing to its extraordinary energy costs, limits the activity toward CO activation. 

### 2.4. Bimetallic

The production of liquid hydrocarbons from carbon monoxide and hydrogen proceeds over the active sites of a FT catalyst. Industrially, Co-based materials are ideal FT catalysts owing to the lower production of organic acid concentrations, whereas metals such as Ru and Ni are not interesting due to a lack of availability or their selectivity [96,97]. One strategy is to alloy metals, resulting in a new underlying electronic band structure, to obtain cheaper, more active, and also highly selective systems in FT reactions. In an earlier work, Ishihara and co-workers studied alloying iron, cobalt, and nickel (Fe–Co, Co–Ni, and Ni–Fe) for CO hydrogenation. They found that alloying leads to the formation of higher hydrocarbons and enhances the adsorption of hydrogen and carbon monoxide [12,23,52]. Especially, new adsorption sites with a new electron density are observed in the case of Co-Ni bimetallic catalysts. They suggested that nickel can be utilized as an activity promoter in low-temperature FTS, and they attributed the effect of alloying on the activity and selectivity for the electronic interactions between the metal species. One of the main characteristics of alloying is to enhance the selectivity toward gasoline. Although iron is selective for the formation of olefins and oxygenates, the formation of a large amount of carbide reduces the catalytic activity. In particular, Co and Ni catalysts are the most active and best candidates for gasoline synthesis, as the former is very selective for the chain growth, and the latter is the most selective for methanation. 

**Figure 20:**
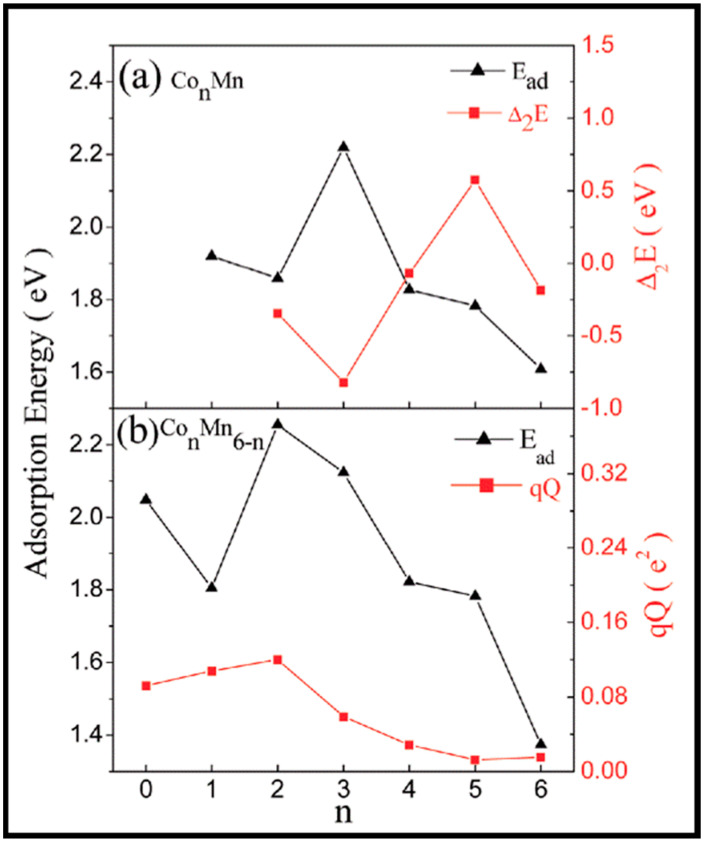
Adsorption energies of (**a**) Co_n_MnCO (*n* = 1–6) and (**b**) Co_n_Mn_6-n_CO (*n* = 0–6). E_ad_ exhibits the adsorption energy, and qQ denotes the strength of electrostatic interaction [98].

It was also suggested that bimetallic clusters with tunable component percentages could be promising candidates for CO and other small molecule adsorption. Du and co-workers studied CO interaction with Co-Mn clusters [92]. The aim of their study was to explore the impact of Co replacement by Mn atoms on the CO hydrogenation procedure. They found that these clusters have high chemical activity, which is desirable for the activation of CO molecules. The calculations seen in Figure 20 display a monotonic decrease in the adsorption energies of Co_n_MnCO, except for *n* = 3, which reveals the strong interaction of CO and Co_3_Mn, which is attributed to the fact that more stable clusters have lower reactivity toward CO adsorption. Moreover, the curve of the adsorption energy reveals that the adsorption capacity follows Co_5_MnCO < Co_4_MnCO < Co_2_MnCO < Co_3_MnCO, which is consistent with the relative stability of the bare clusters (Co_5_Mn > Co_4_Mn > Co_2_Mn > Co_3_Mn). However, although the Co_5_Mn cluster is exceptionally stable, its adsorption energy by the CO molecule is larger than that of Co_6_Mn, owing to a different adsorption pattern where the CO molecule is adsorbed in the hollow site on the surface of the Co_5_Mn cluster but atop the Co atom of the Co_6_Mn cluster; the former leads to more metal–carbon bonds than the latter and, thus, induces stronger binding of the CO molecule [98].

**Figure 21:**
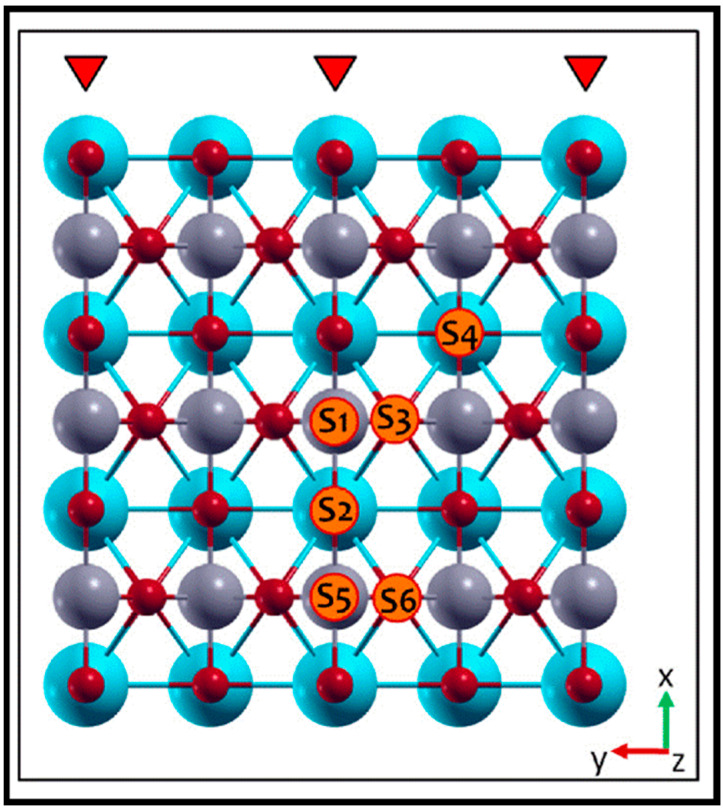
The illustration of calculated CO adsorption sites from top view [77].

The adsorption characteristics of CO on pure and Co-doped SrTiO_3_ were investigated by Carlotto and co-workers using DFT calculations [77]. They considered several distinct adsorption sites for both doped and un-doped systems, as depicted in Figure 21. The results show that CO preferentially adsorbs on the step–edge oxygen adjacent to the Ti atom on both pure and doped surfaces. Furthermore, the adsorption of CO on the S1 site (Ti atom at the step–edge) of the pure surface is more favorable than at the doped surface, which is in contrast to the S3 site (oxygen atom at the (100) terraces). 

**Figure 22:**
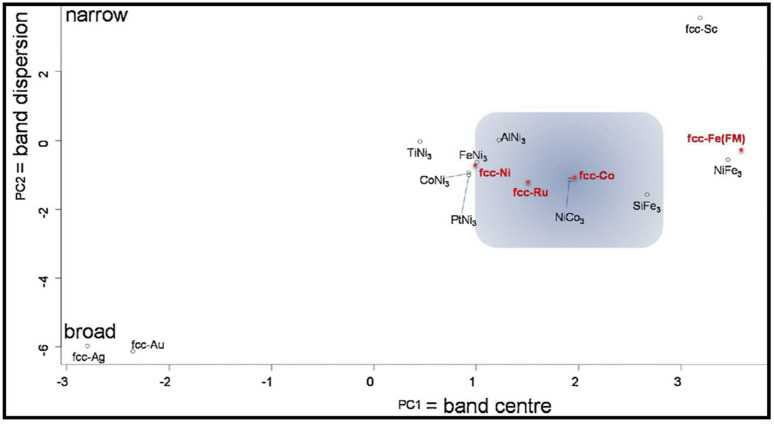
Biplot along the dominant principal axes, for which the vertical axis relates to d-band dispersion, and horizontal axis denotes d-band center. The blue color zone shows the systems with same activity as Fischer–Tropsch active metals [29].

The commercial focus of bimetallic catalysts for low-temperature Fischer–Tropsch (FT) synthesis is mostly on the Fe- or Co-based systems, as Ni-based systems suffer from poor selectivity. However, Helden and co-authors prepared and examined alternative bimetallic catalytic materials, containing both Co and Ni in different ratios, and studied the materials’ activity and selectivity for FT synthesis [29]. Hence, the adsorption strength of oxygen and carbon atoms on the proposed surfaces is considered as comparable to the Co surface as a baseline. The final chosen alloys, based on a low cost and similar C and O adsorption properties as Co, are SiFe_3_, AlNi_3_, FeNi_3_, NiFe_3_, FeCo_3_, and NiCo_3_. ZnNi, ZnCo_3_, CuCo_3_, GeFe_3_, GaFe_3_, ZnFe_3_, AsFe_3_, RuCo_3_, PdCo_3_, GaRu_3_, PtCo_3_, RhCo_3_ alloys are almost as good, but they are expensive. Among the above-mentioned alloys, Ni catalysts are known as large methane producers, but the electronic structure parameter results plotted in Figure 22 exhibit that NiCo_3_ alloy has a very similar behavior as Co. As such, they mainly focused on Co-Ni alloy synthesis and the analysis of catalytic properties for varying Ni ratios. They found that Co-Ni alloys are easily formed and are cheaper than cobalt, up to a nickel content of 25%, and these alloys perform as well as Co during the FTS process for CO conversion. 

**Figure 23:**
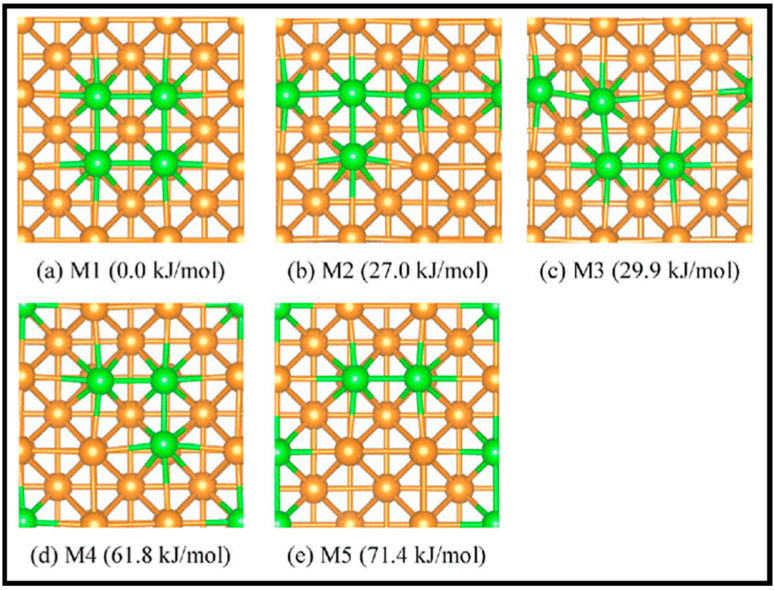
Different arrangements of four Co atoms embedded in the first layer of Cu (100) surface from top views, with their relative energies shown in parentheses. Orange and green spheres stand for Cu and Co atoms, respectively [76].

Recently, the hydrogenation of CO was explored on a modified Cu (100) surface by introducing Co nano-clusters into the top layer [76]. Qiu et al. found that the strong Co-O adsorption bonds in the modified surface enhance the stability of the formaldehyde intermediate. Moreover, the addition of the Co_4_ cluster to the surface also improved the selectivity toward methanol on the Cu (100) surface. Figure 23 shows the most stable structures of the Co_4_/Cu (100) surfaces (M_1_~M_5_), where the M_1_ model is energetically preferred. Depending on which atom of the molecule interacts with the H* atom, four possible routes exist to form CH_3_OH, as shown below:

Path 1: CO* → COH* → HCOH* → H_2_COH* → H_3_COH* 

Path 2: CO* → HCO* → HCOH* → H_2_COH* → H_3_COH* 

Path 3: CO* → HCO* → H_2_CO* → H_2_COH* → H_3_COH* 

Path 4: CO* → HCO* → H_2_CO* → H_3_CO* → H_3_COH* 

**Figure 24:**
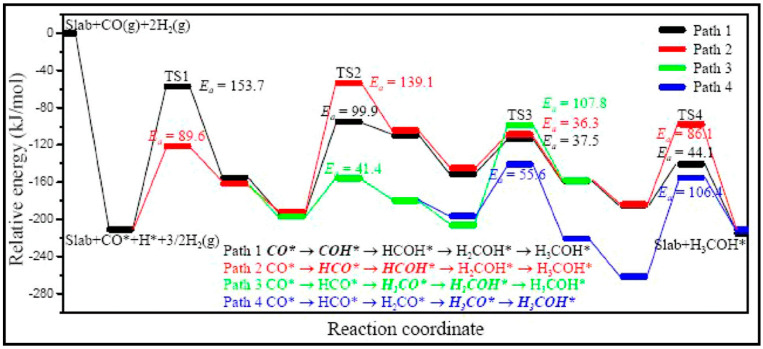
The overall potential energy profile via CO* hydrogenation on Co_4_/Cu (100) surface to form CH_3_OH [76].

It is assumed that atomic H* is available during all steps of CO hydrogenation. The potential energy of the different pathways is presented in Figure 24. According to the energy profile, the rate-limiting steps in the four paths is the CO* + H* → COH* reaction during the O−H bond formation; the hydrogenation of HCO* (HCO* + H* → HCOH*); the H_3_* attack of H_2_CO* to create a new O–H bond; and the breaking of the Co–O adsorption bond during the hydrogenation of the H_3_CO* species, in paths 1, 2, 3, and 4, respectively. Overall, the fourth pathway is dominant in the production of methanol, while the others possess relatively high energy barriers. So the elementary steps for CO* conversion to methanol are as follows:

$${\mathrm{CO}}^{*}\overset{{+H}^{*}}{\to }{\mathrm{HCO}}^{*}\overset{{+H}^{*}}{\to }H_{2}{\mathrm{CO}}^{*}\overset{{+H}^{*}}{\to }H_{3}{\mathrm{CO}}^{*}\overset{{+H}^{*}}{\to }H_{3}{\mathrm{COH}}^{*}$$

Furthermore, O–H bond formation is the rate-limiting step in all four pathways, revealing that hydrogenation through the oxygen site needs to overcome a higher energy barrier compared to hydrogenation through the carbon site. 

**Figure 25:**
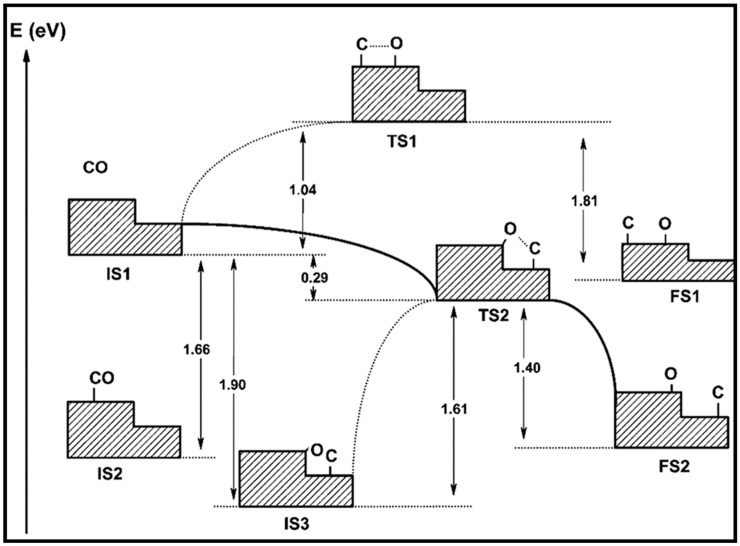
The CO dissociation energy profile on both flat and stepped Co (0001). IS_1_, IS_2_, and IS_3_ are the initial states with CO in the gas phase, the chemisorption state with CO on flat Co (0001), and the chemisorption state with CO on stepped Co (0001), respectively. TS_1_ indicates the transition state of CO dissociation on flat Co (0001), while TS_2_ displays the transition state of CO dissociation between the step–edge and the terrace below on stepped Co (0001). Two final states (FS_1_ and FS_2_) are CO dissociation with O and C chemisorbed on flat Co (0001) and on the step–edge and the terrace on stepped Co (0001), respectively [73].

One of the major intermediates in the FT process is the O atom, which can poison the catalyst, so its removal through hydrogenation is, therefore, of crucial importance. Hu et al. considered CO dissociation and O removal on flat and stepped Co (0001) surfaces [73]. The calculated adsorption of C, O, H, OH, and CO revealed the similar chemisorption energies of H in contrast to the different chemisorption energies of other species on the distinct sites of the flat and stepped surfaces. They suggested the step–corner site as the most stable for CO and C chemisorption, but the highest chemisorption energy for the OH group was found on the edge–bridge site of the stepped surface. They also identified the low likelihood of O or OH chemisorption on the step–corner site, where the O or OH group moves away from the initial step–corner to the terrace after optimization. The adsorption and dissociation of CO on flat and step surfaces are compared and reported in Figure 25. CO dissociation takes place through the direct channel on the stepped surface, and, in general, defects are the most suitable sites for CO dissociation to occur. As for O removal through the formation of water, they demonstrated that the first step of hydrogenation, O + H → OH, is more facile on the stepped surface than the flat surface, since the barrier is significantly reduced, Table 17. In contrast, further hydrogenation to produce water is not more likely on the stepped surface with respect to the flat plane, but it is difficult to achieve on either surface at low coverages. 

## 3. Non-Cobalt Catalysts

Cheng and co-authors investigated other commonly used catalysts for FTS, such as Fe, Rh, and Ru [99], and compared them to the Co studies in their previous work [77,99]. The C–C coupling reactions and relative stabilities of CH_i_ (i = 1–3) are calculated on the stepped metal surfaces, since they are active for coupling and breaking the C–C chain. The relative stabilities of CH_i_ (i = 1–3, E_i_) species are defined by the total energy difference, in which the total energy of the C + 4H adsorbed on the surfaces is subtracted from the total energy of the CH_i_ + (4–i)H adsorbed on the surface. Thus, a larger E_i_ value means that the species have lower stability. As shown in Table 18, C and CH are stable, with C being the most stable in contrast to the unstable CH_2_ and CH_3_ species. Overall, CH stability does not considerably vary on various catalyst surfaces, whereas the relative stability of CH_2_ and CH_3_ increases by the metal activity. The chemical activity of these metals toward CH_4_ was measured and is also listed in Table 18. The extent of the negative value indicates the strength of bonding and the activity of the metal surface; the more negative they are, the more active the surface is. The authors found that the reaction rate of each C_1_ + C_1_ coupling pathway can be evaluated by the summation of the barrier of the coupling reaction and the stabilities of the reactants, E_i_,_j_ + E_i_ + E_j_. As is clear from Table 19, the major chain-growth pathways are different on each metal: C + CH and CH + CH coupling on Rh and Ru; C + CH_3_ and CH_2_ + CH_2_ coupling on Co; and C + CH_3_ coupling on Fe, with the fastest coupling pathways shown in bold. These different pathways can be ascribed to the variation in the relative stabilities of C_1_ species and the coupling barriers on different metals. An unexpected result is that all the major chain-growth routes on the surfaces include C + CH and/or CH + CH coupling, since C and CH are more stable on these surfaces. 

### 3.1. Iron

**Figure 26:**
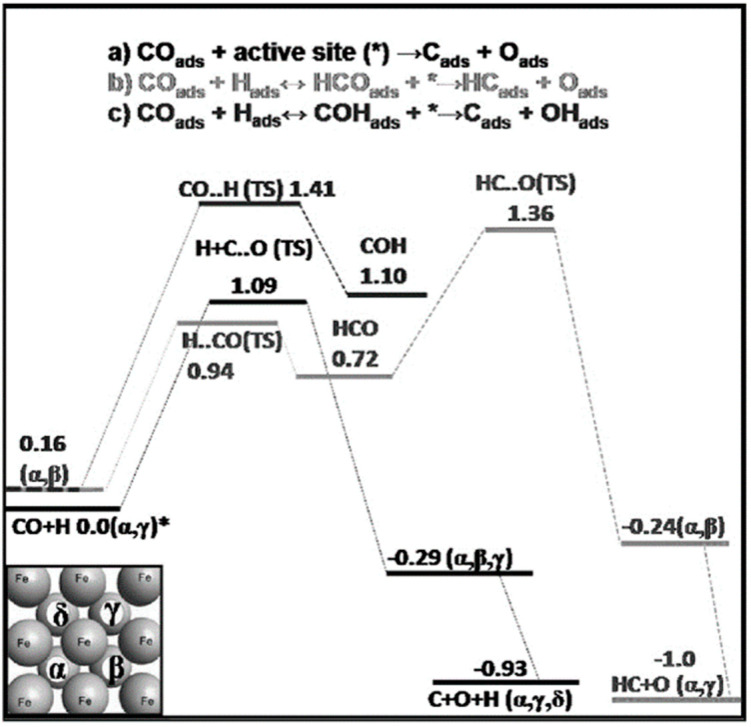
The relative energy in eV and CO dissociation pathways via (a) direct CO dissociation, (b) via HCO intermediate formation, and (c) via COH intermediate formation. The structure model is presented in inset, which shows the hollow sites available for co-adsorption [62].

Iron-based catalysts attracted attention for FTS due to their low price, high activity, and low CH_4_ selectivity [100,101,102]. The direct and H-assisted CO dissociation on the Fe (100) surface were investigated and compared. It was shown by Elahifard et al. that both mechanisms are energetically feasible [62], and the energies of the most stable adsorption modes are plotted in Figure 26. They found that the dominant mechanism for CO dissociation on Fe (100) is dependent on the conditions. Although H-assisted CO dissociation via HCO is not very efficient in comparison to direct CO dissociation, the H-assisted CO dissociation route via HCO is most likely under high H_2_ pressure and low temperatures. Their studies also revealed the negligible effect of H co-adsorption on CO and the low activation energy for the direct process in the absence of hydrogen. Furthermore, they indicated that empty sites are created through HCO formation (path b sketched in Figure 26), while active sites are consumed during direct CO dissociation. However, despite the decrease in active sites, which stops the direct route, path b suffers from a reduction in conversion efficiency. The adsorption and direct dissociation of CO on the Fe (100) surface in the presence of hydrogen was also explored in the work by Roncancio and co-authors [103]. Their calculations indicated the positive influence of hydrogen pre-adsorption on CO adsorption, leading to a lower dissociation energy barrier (see Table 20), which is attributed to the major transference of electronic density from the Fe surface to the adsorbed CO, causing a covalent interaction between Fe and C. They also confirmed the reported result of the small effect of hydrogen co-adsorption on the activation barrier for CO dissociation. In another work, the oxygen-terminated a-Fe_2_O_3_ (0001) surface was studied for carbon monoxide (CO) adsorption by Xiao and co-workers [104], who found that since there are no active sites on the Fe_2_O_3_ surface, the CO does not preferably adsorb on the surface at low CO pressure. Oxygen vacancies are created during the oxidization of CO to form a bent CO_2_ dimer, followed by a reaction with dissociated water to produce a carboxyl group. 

**Figure 27:**
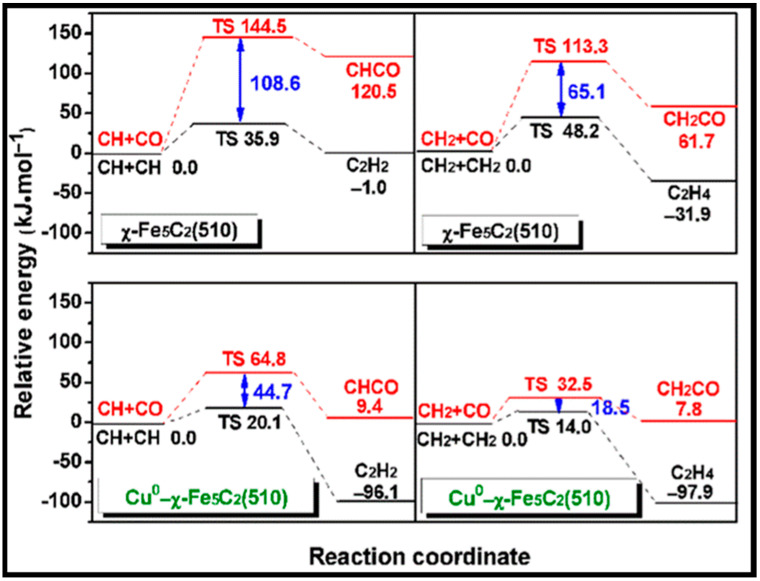
The potential energy profiles of (left panels) CH_x_ + CH_x_ (x = 1, 2) coupling and (right panels) CO insertion into CH_x_ (x = 1, 2) species on χ-Fe_5_C_2_ (510) and Cu^0^–χ-Fe_5_C_2_ (510) surfaces, respectively [106].

The presence of promoters in iron-based Fischer–Tropsch catalysts facilitates the reduction of trivalent iron to divalent iron during catalyst activation [99]. Here, the role of copper in the iron-rich χ -Fe_5_C_2_ (100)_0_._25_ surface and binary Cu^0^–χ-Fe_5_C_2_ (510) catalyst is introduced. Steen and co-workers considered the impact of a single copper atom on the surface of the Hägg iron carbide on the bonding of CO during adsorption at the surface [105]. The co-adsorption of copper (in its most stable position) and carbon monoxide on the χ -Fe_5_C_2_ (100)_0_._25_ surface limits the access by CO to the surface. At low CO coverage, the existence of copper as a chemical promoter leads to the elongation of the C–O bond and enhances the bonding of CO to the surface, although copper only acts as a chemical promoter for a short time. Since binary metal catalysts are equally active, and selective catalysts are composed of Earth-abundant metals, they seem promising candidates for the replacement of precious metals. Lu et al. investigated the CO hydrogenation reaction on the Cu–Fe binary catalyst in the production of higher alcohols (C_2_ + OH) [106]. A comparison of the reaction profiles of CH*_x_*+ CH*_x_*(*x* = 1,2) coupling and CO insertion into CH*_x_*(*x* = 1,2) reactions for both Cu^0^–χ-Fe_5_C_2_ (510) and χ-Fe_5_C_2_ (510) is sketched in Figure 27. As can be seen, the activation barriers for the formation of C_2_-hydrocarbons (C_2_H_2_/C_2_H_4_) and C_2_-oxygenates (CHCO/CH_2_CO) over the Cu^0^–χ-Fe_5_C_2_ (510) surface are significantly lower than those on χ-Fe_5_C_2_ (510). Hence, higher catalytic activity is achieved by Cu^0^–χ-Fe_5_C_2_ (510) toward the formation of C_2_-hydrocarbons and C_2_-oxygenates, with respect to χ-Fe_5_C_2_ (510). Furthermore, the activation barrier difference suggests more selectivity toward C_2_-oxygenates on Cu^0^–χ-Fe_5_C_2_ (510), ascribed to the closer d-band center of the Cu^0^–χ-Fe_5_C_2_ (510) surface to the Fermi level and the electron-rich interface of Cu^0^–χ-Fe_5_C_2_ (510), which promotes CO activation and CO insertion into alkyl species. The proposed reaction pathway for CO hydrogenation on Cu^0^–χ-Fe_5_C_2_ (510), starting from the CHCO intermediate, is, thus:

CHCO + (H) → CH_2_CO + (H) → CH_3_CO + (H) → CH_3_CHO + (H) → CH_3_C H_2_O + (H) → C_2_H_5_OH

Recently, the activity and selectivity of two highly active iron- and cobalt-based catalysts were studied by Davis et al. [107]. They illustrated that cobalt catalysts have a much higher initial CO conversion rate than Fe ones. As is apparent from Table 21, it was also found that Co prefers to produce heavier hydrocarbons, while Fe is selective toward CH_4_ and olefins. However, increasing CO conversion leads to a reduction in the CH_4_ selectivity and an increase in the C_5+_ selectivity. For CO conversion greater than 80%, Co rapidly deactivates due to cobalt oxidation, but iron shows stability over the entire CO conversion range. 

### 3.2. Nickel

**Figure 28:**
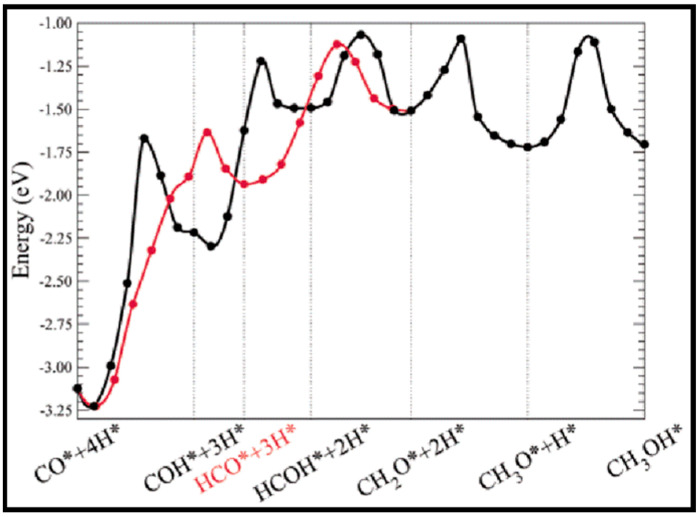
The reaction energy path for methanol formation on Ni (111). * Denotes adsorbed species [114].

**Figure 29:**
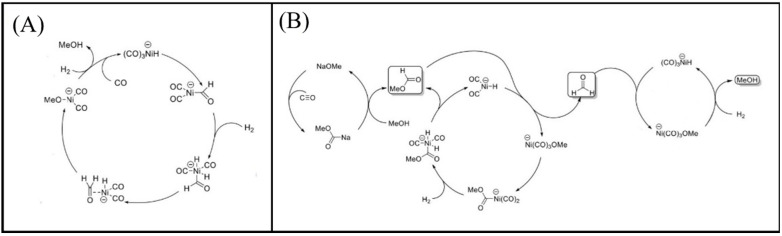
(**A**) The proposed direct hydrogenation mechanism of CO to produce methanol. (**B**) The possible mechanism for indirect (via methyl formate) hydrogenation of CO to MeOH [115].

Among the Cu, Co, and Ni catalysts for CO hydrogenation, Cu catalysts highly favor methanol production, while Co catalysts strongly favor methane formation [5]. Although methanol synthesis is crucial in the fuel industry, some experimental studies suggested that CO does not contribute to methanol formation on Cu-based catalysts [108], whereas Ni was suggested as a highly active methanation catalyst that dissociates CO to form methane [109]. In other words, the theoretical and experimental results proposed Ni catalysts to be active toward methanation as well as methanol formation [110,111,112,113]. Hence, Remediakis et al. explored the synthesis of methanol from CO and H_2_ on a Ni catalyst [114]. They indicated that in the co-adsorption of CO and H on Ni (111), CO hydrogenation is mostly favored over the dissociation or desorption of CO. Furthermore, as shown in Figure 28, the highest calculated reaction energy of transition states through to methanation is lower than the energy of the gas-phase reactants. This result implied the applicability of cheap Ni catalysts for methanol formation from CO and H_2_. Later, McGuinness et al. considered two different mechanisms of methanol synthesis (CO + 2H_2_ → CH_3_OH) on the Ni catalyst surface [115]. They compared the possibility of direct (via formyl intermediates) and indirect (via methyl formate) routes of CO hydrogenation for methanol production, as shown in Figure 29. The results revealed the feasibility of methanol formation through both direct and indirect routes, but the former has no significant participation in methanol production at low temperatures, while the latter has a much higher activation barrier. A combined theoretical and experimental investigation of CO hydrogenation on a Ni (110) surface was performed by Ashwell and co-authors [5], who found that the production of methanol by the sequential hydrogenation of CO is energetically favorable. They also experimentally confirmed the formation of methanol and formaldehyde from CO hydrogenation on Ni (110). It was shown that the hydrogen subsurface varies the electronic structure of the metal surface, which leads to an increase in the adsorption energy and methanol production. CO hydrogenation requires a lower activation energy than CO dissociation, with or without the hydrogen subsurface [5].

Nickel-based catalysts are known as promising water–gas-shift (WGS) reaction catalysts, which is a key step in the generation of hydrogen from carbon-based materials in industry. The mechanism of the WGS reaction on Ni (111) surfaces was studied to understand the effects of CO and H_2_O adsorption on the activation energy of the rate-determining reactions [41]. Three elementary reactions, named WGS I and WGS II, according to the formate intermediate, and WGS III, based on direct oxidation, were investigated [41]. In the WGS I model, the direct reaction between adsorbed water and carbon monoxide leads to the formation of formate, whereas, in the WGS II mechanism, H_2_O dissociates to OH and H, and then the adsorbed carbon monoxide directly reacts with the adsorbed OH. The WGS III mechanism is based on the direct oxidation of carbon monoxide, in which CO reacts with the water dissociation product (O). The direct pathway is preferred on the Ni (111) surface, but it should be mentioned that adsorbed water and carbon monoxide on the nickel surface show a zero-energy reference. The results reveal that the formate intermediates, which involve the reaction between adsorbed H_2_O and CO species, are the best reaction routes in the WGS mechanism [41].

### 3.3. Ruthenium

**Figure 30:**
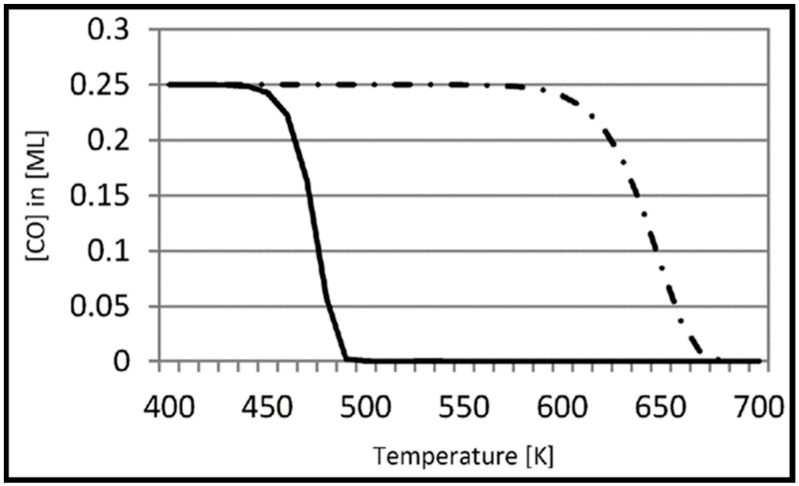
The full line is related to the competitive reaction of CO dissociation and CO desorption on Ru (0001). The dotted line shows the desorption of CO by itself at the initial coverage of 0.25 ML [2].

**Figure 31:**
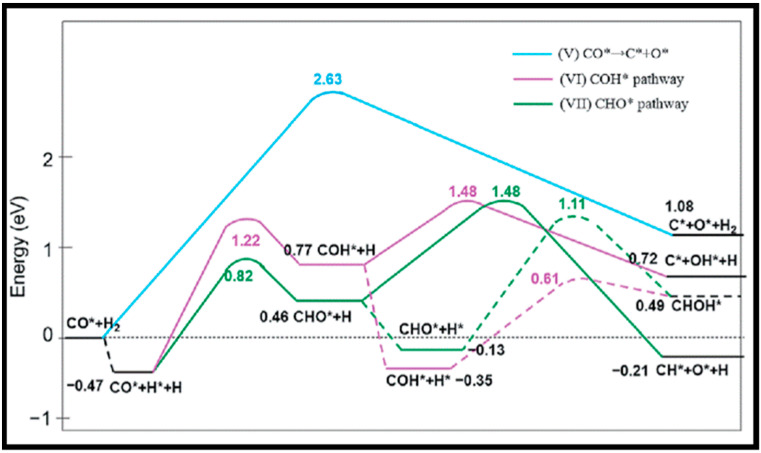
Energy profile dissociation pathway through direct and H-assisted pathways on Ru (0001) surface. The summation of adsorbed CO and H_2_ adsorption energies are considered as reference [1].

**Figure 32:**
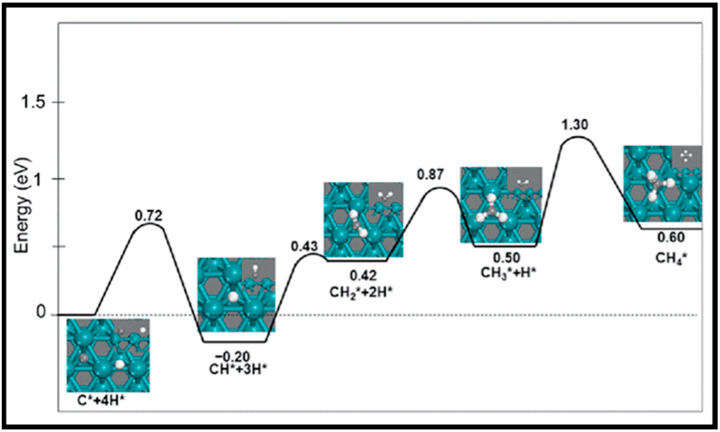
The reaction energy profile for hydrogenations of carbon to CH, CH_2_, CH_3_, and CH_4_ on the Ru (0001) surface [1].

Mirwald et al. studied CO dissociation and the hydrogenation mechanism on the Ru (0001) surface [2], finding that insertion is the preferred CO conversion route rather than the carbide mechanism. However, when CO desorption was considered versus CO dissociation, the results, as reported in Table 22 and Figure 30, reveal the opposite. According to the activation barrier values reported in Table 22, one can deduce that desorption is favored over dissociation, as also found on the Co catalyst, whereas microkinetic simulations suggest the unexpected result that carbon monoxide dissociates rather than desorbs. It should be mentioned that desorption is favored at low temperatures, but the process is too slow. Furthermore, the CO reaction with CH_x_ is explored via the migration of the methylene/methylidyne species toward CO and the insertion of CO into the Ru–C bond. As seen in Table 22, the higher activation barrier of CO dissociation compared to insertion and migration indicates that dissociation competes with both insertion and migration in the presence of hydrocarbon fragments, and, overall, the migration mechanism is the main reaction pathway on Ru (0001). Zhang and co-workers also investigated the hydrogenation mechanism on the Ru (0001) surface [1], demonstrating that CO hydrogenation through both COH and CHO intermediates produces active C and CH species, with further hydrogenation leading to the formation of CH_4_ or longer carbon chains. The energy profiles of CO dissociation and hydrogenation, as presented in Figure 31, illustrate that direct CO dissociation is less favorable than dissociation via COH and CHO intermediates. Owing to higher stability, CO hydrogenation likely forms C and CH intermediates on Ru (0001). Moreover, it was shown that the C–OH bond breaks more easily than the HC–O bond on the Ru (0001) surface. The results display the weak interaction of intermediates with the Ru (0001) surface and small adsorption energies, revealing that intermediates can easily escape from the surface. The carbon hydrogenation energy profile to form methane, as exhibited in Figure 32, indicates CH_4_ formation from CH_3_ as the rate-limiting step, since it requires the highest activation energy. C–C coupling reactions on the Ru (0001) surface were also examined, showing a high barrier for the reaction between all CH_x_ species and CO and a low one via CH + CH. Hence, the chain growth between CH_x_ species and CO is not likely to occur, but C–O bond cleavage is favorable on the Ru (0001) surface. The two most desirable reactions, CH + H and CH + CH, compete with each other to produce methane and liquid hydrocarbons, respectively. The former occurs at a high H_2_ fraction and normal pressure, while the latter is favorable at a high CO fraction and pressure. 

**Figure 33:**
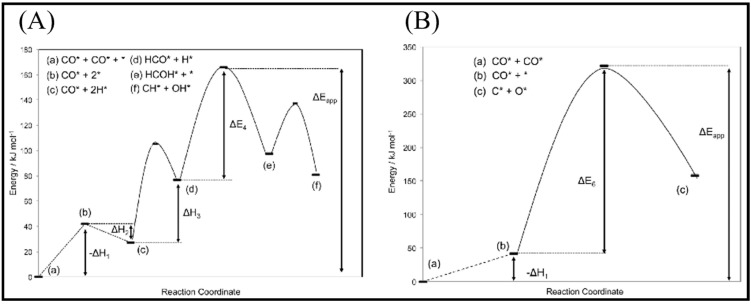
(**A**) Energy profile for elementary steps through H-assisted CO* activation path on the (111) terrace of Ru (201( (1.55 ML CO*). The barrier of the HCOH* dissociation to CH* + OH* ((e) to (f)) justifies the irreversibility of HCO* hydrogenation. (**B**) Energy diagram for direct CO* activation on (111) terraces of Ru (201) (1.55 ML CO*). The apparent activation energy (ΔE_app_) is the summation of the required energy to generate a vacancy (−ΔH_1_) from a CO*-covered surface and direct activation energy of CO* to form chemisorbed C* and O* species [110].

The effect of CO* coverage and C−O bond activation on Ru (0001) and Ru (201) clusters was explored by Loveless et al. [116], who showed that the weakening of binding due to increasing coverage occurs at higher coverages on Ru (201) clusters than on Ru (0001) surfaces (CO*/Ru = 1.55 to 0.75), which is ascribed to the weaker repulsive interactions on the surface of small Ru (201) clusters. CO* activation predominantly occurs via the H-assisted route for CO* at the (111) terraces of Ru (201) cluster surfaces, whereas direct CO dissociation is not desirable on Ru atoms under saturated CO* coverage conditions. Figure 33 and Table 23 present the activation energies and reaction profiles of H-assisted and direct CO* activation on the (111) terraces of Ru (201) clusters. The addition of hydrogen to the carbon atom of CO* is the main hydrogenation route, rather than the unlikely direct activation path. Since the corner and edge atoms are not active, the existence of vacant sites is required for both activation paths, but the dissociation of CO at vacant Ru sites requires larger activation barriers than the H-assisted CO dissociation on the (111) terraces. Recently, CO activation through direct and hydrogen-assisted routes on the terraces and step–edges of Ru surfaces was explored by a molecular dynamics simulation [117]. The results suggested the hydrogen-assisted CO activation mechanism on the step–edges is the favored mechanism at high CO coverage. In addition, the size of nanoparticles (NPs) was considered, and it was found that the step–edges of small NPs have lower reactivity than those on large NPs in the COH* formation. The hydrogen transfer to CO on the flat Ru surface is promoted without weakening the C–O bond, which leads to the formation of formyl intermediates (HCO*) and formaldehyde. In contrast, the facile hydrogen transfer on the step–edges of Ru surfaces causes C–O bond cleavage and, consequently, the direct formation of C* and OH* or indirect CH* and OH* production. Generally, hydrogen-assisted pathways on the step–edges of Ru surfaces are most desirable and activate CO cleavage. 

**Figure 34:**
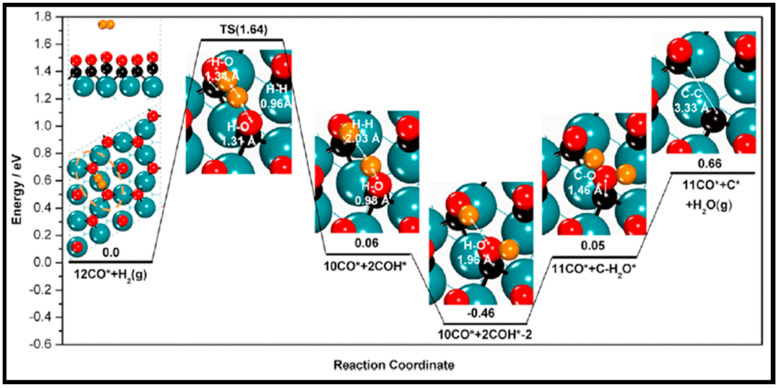
The energy diagram including IS, TS, and FS of all structures involved in the CO self-promoting hydrogenation path on CO*-saturated Ru (0001) (surface Ru/blue; C/black; O/red; H/yellow) [118].

**Figure 35:**
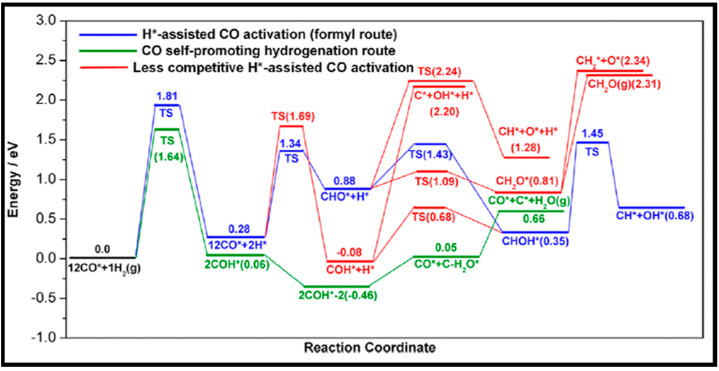
Energy diagram for three CO activation routes on CO-saturated Ru (0001) [118].

The co-adsorption of two adjacent CO* as well as CO and H_2_ co-adsorption were studied on Ru (0001) by Zhao et al. [118]. They found that two adjacent co-adsorbed CO* behave as promoters during the bond scission of the H–H bond and the formation of two COH* molecules. This CO self-promoting hydrogenation route is shown in Figure 34. As can be seen, the direct reaction of adsorbed C* with H_2_ occurs after H_2_O removal to generate CH_2_* according to the chain-growth process. This CO self-promoting hydrogenation route to form CH_2_ ($CO^{*}+CO^{*}\overset{+H_{2}}{\to }\underset{-H_{2}O}{\to }C^{*}+CO^{*}\overset{+H_{2}}{\to }CH_{2}^{*}+CO^{*}$) resembles the carbide mechanism ($CO^{*}\overset{+H_{2}}{\to }\underset{-H_{2}O}{\to }C^{*}\overset{+H_{2}}{\to }CH_{2}^{*}$), where both routes produce hydrocarbons rather than oxygenated products. In the case of hydrogen and carbon monoxide co-adsorption, the H_2_ molecule dissociatively adsorbs on CO-saturated Ru (0001), which is neither kinetically nor thermodynamically desired. Figure 35 shows the reaction profiles of different hydrogenation paths and reveals the formyl (HCO*) route as the most favorable in H*-assisted CO activation. The irreversible reactions of HCO* with H*, followed by HCOH* dissociation, lead to the formation of CH* and OH*.

**Figure 36:**
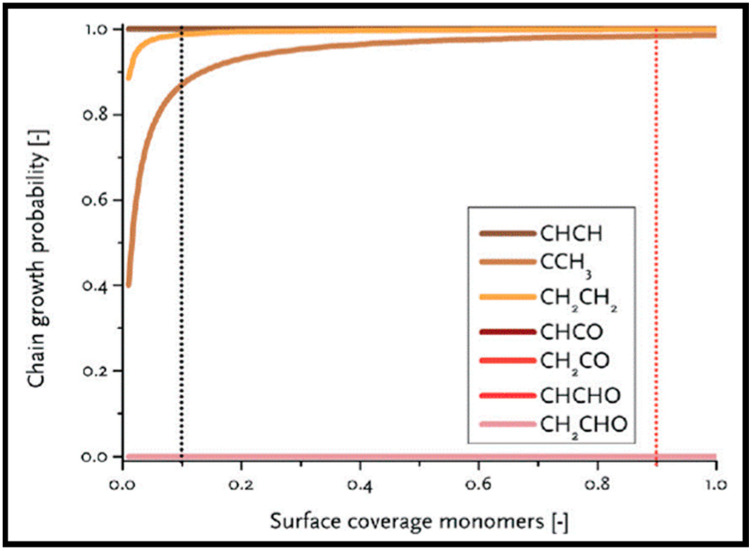
The chain-growth probability as a function of surface coverage. The red and black dotted lines are representative of C_1_ and CO coverage [119].

**Figure 37:**
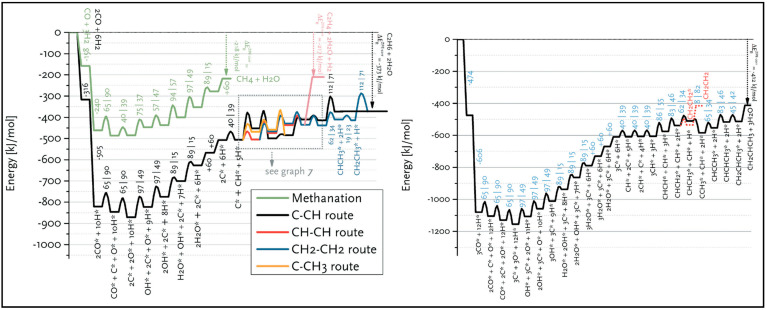
Reaction energy diagram for (**left**) ethane, ethylene, and methane and (**right**) propylene formation from carbon monoxide and hydrogen [119].

**Figure 38:**
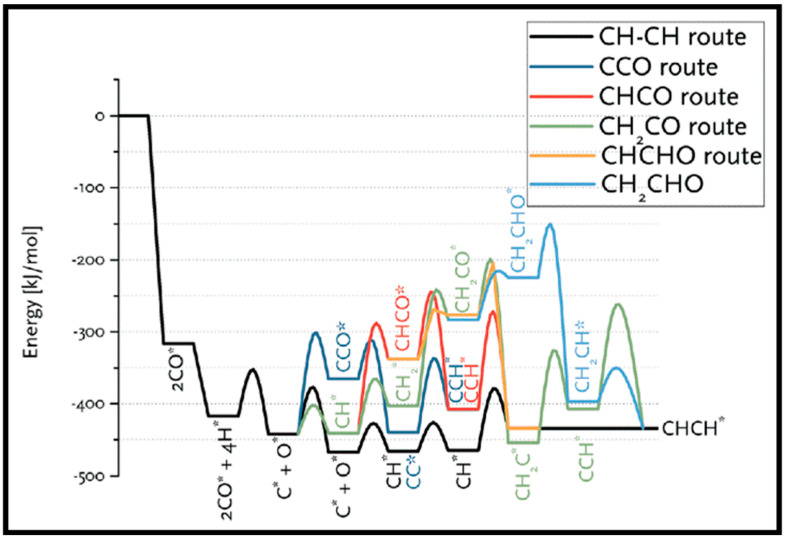
Reaction energy profile for CHCH* formation, where various routes are compared to the CH–CH route in the carbide mechanism [119].

In addition to the Ru (0001) surface, the carbide and CO insertion mechanism were investigated on the Ru ($11\bar{2}1$) surface by Filot et al. [113]. They found that the chain growth in the carbide mechanism along the preferred pathway of the CH + CH coupling is more favorable than the CO insertion mechanism along the preferred pathway of the C + CO coupling and methane production. Also, their results indicated that the chain growth on the step–edge is more facile than that on the terrace sites. The rate of the fast chain growth versus the rate of the chain-growth termination is an important factor in the production of long hydrocarbon chains in both mentioned mechanisms, and the rate constants for the most desirable routes are reported in Table 24. Since surface coverage plays a role in the chain growth, the probability of the chain growth against the surface coverage for the reported routes in Table 24 is shown in Figure 36. As is clear, at all CO coverages the chain-growth probability is negligible in the case of CO insertion, in contrast to the higher-than-zero probability of the three pathways to the chain growth in the carbide mechanism. Hence, the preferred route of methane formation is CH_x_ hydrogenation, rather than chain propagation with CO. Furthermore, the reaction energy profiles of methane, ethane, ethylene, and propylene formation from carbon monoxide and hydrogen in the carbide mechanism are depicted in Figure 37. Although C and CH are the most stable intermediates, and CH is also the dominant chain-propagation intermediate, their coupling reaction is unfavorable, whereas the coupling of two CH_2_ fragments is facile but endothermic. Moreover, C + CH_3_ coupling is unfavorable, since the formation of CH_3_ is endothermic, and the coupling barrier of C is high. Figure 38 illustrates the pathways toward CHCH* formation through the CO insertion mechanism. The growing chain is initiated from adsorbed C* via CO dissociation, i.e., the same as the carbide mechanism. The coupling of carbon with CO, which is followed by CC formation and then hydrogenation, is the most desirable path. As in the carbide mechanism, C and CH are the most stable surface intermediates, while CH_2_ shows lower surface stability, with a slightly higher coupling barrier for CH_2_ + CO relative to that of C and CH. As is apparent from Figure 36, Figure 37 and Figure 38, CH + CH coupling in the carbide mechanism has lower overall barriers in comparison to the CO insertion mechanism. 

**Figure 39:**
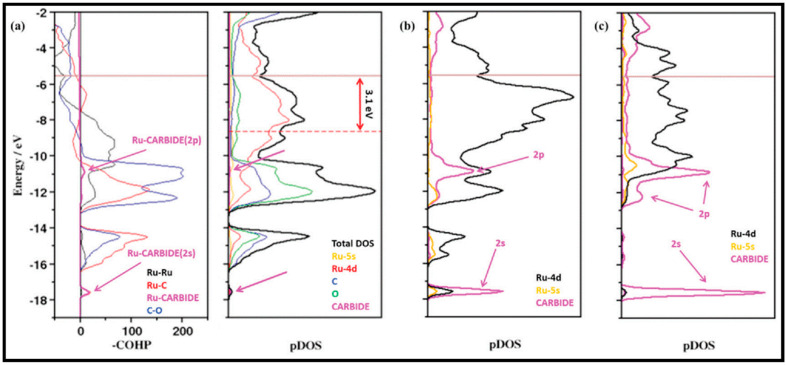
(**a**) pCOHP(ε) and pDOS(ε) profiles for intermediate. (**b**) pDOS(ε) profile highlighting the interaction of the surface carbide with neighboring in-plane surface Ru atoms and (**c**) the vicinal core Ru atom [120].

Although the formation of ruthenium carbides during CO bond cleavage was extensively reported, their easy formation under mild conditions at small Ru nanoparticles (RuNPs) during CO hydrogenation needs further exploration. Moraru and co-workers investigated the formation of stable carbides during CO bond dissociation [120]. Their calculations revealed that carbide forms via hydrogen-assisted hydroxymethylidyne (COH) pathways within a reasonable kinetic cost on various sites of RuNPs. They also indicated the possibility of the formation of μ6 ruthenium carbides at the B_5_ site of Ru NPs, just like molecular ruthenium clusters. The signature of carbide in intermediates is plotted in the projected density of states (pDOS) and crystal orbital Hamilton population (pCOHP) profiles (see Figure 39). The interaction between the carbide and neighboring surface ruthenium atoms, as shown in Figure 39b, is stronger than the interaction between the carbide and the proximal core ruthenium atom (Figure 39c). As is clear from Figure 39c, the further adsorption of a CO or H_2_O molecule in the vicinity of the ruthenium carbide enhances the adsorption of the carbide to the core Ru atom. Direct CO dissociation is another way toward carbide formation, but with a higher activation energy and lower probability compared to the H-assisted route. 

### 3.4. Rhodium

The production of ethanol as a renewable energy source and intermediate in the formation of light olefins has received significant attention. Ethanol is one of the major products during CO conversion on Rh surfaces as well as olefins and oxygenates (mainly methanol, acetaldehyde, and acetic acid) [40]. In a pioneering work, Mei et al. examined Rh-based/SiO_2_ catalysts and the impact of alloying with several promoters (Mn, Ir, Ga, V, Ti, Sc, Ca, and Li) on the selectivity toward ethanol [42], and they found that, in alloying the promoters, the difference in electronegativity plays a crucial role in the CO insertion mechanism, which lowers the reaction barriers and results in high selectivity toward ethanol [42]. However, Mei and co-authors only provided a brief description of the reaction energies and activation barriers of the major reaction intermediates and methanation reactions on Rh and Rh/Mn nanoparticles. Two Rh/Mn clusters with Mn surface compositions of 10% (Rh_49_Mn) and 33% (Rh_47_Mn_3_) were studied, and they showed that the internal Mn atoms of Rh/Mn nanoparticles do not significantly affect the calculated energies. Doping with one Mn atom leads to a slight increase in methane formation, while the addition of three Mn atoms lowers the methanation barrier by a moderate amount. Thus, it can be inferred that Mn doping has no significant effect on the activation barrier in methane production. It should be noted that methane formation is still inevitable in the CO hydrogenation process, even in the presence of the Mn promoter. In contrast to the activation energy barriers in methane formation, Mn doping lowers the barrier to CO insertion into CH, although the activation barriers are still high for CO insertion into CH_2_ and CH_3_. CO insertion into CH species is not favorable on pure Rh_50_ and Rh_49_Mn, where it competes with methane formation on the Rh_49_Mn and Rh_47_Mn_3_ nanoparticles. As such, high concentrations of Mn are required in the Rh/Mn alloy to provide a feasible promoted pathway for CO insertion into CH. Moreover, the insertion of CO into CH_2_ and CH_3_ is difficult, even with the addition of Mn into Rh, which causes the high selectivity toward methane formation in the presence of Mn promoters. According to Mei et al. [42], the CO + CH route is the preferential pathway for CO insertion among the three CO insertion routes. As a result, the CHCO intermediate is the essential reaction intermediate in the formation of oxygenates. Temperature also has a major effect on the selectivity toward different products, due to variation in the H coverage [42]. Overall, the ethanol selectivity increases with an increase in temperature, while the selectivity toward other products decreases. 

Filot et al. also studied the reaction energies for the conversion of CO into methane, ethylene, ethane, formaldehyde, methanol, acetaldehyde, and ethanol [40]. Since the CO dissociation barrier is high on the Rh terrace surfaces, step–edges were also considered in the CO hydrogenation process. It was found that the CO dissociation barrier on the Rh (211) surface is much lower compared to that on the Rh (111) and Rh (100) terraced surfaces. Direct CO dissociation is the dominant pathway for CO activation on Rh (211) compared to the H-assisted CO dissociation route. The investigation of the elementary reaction steps and product selectivity as a function of temperature showed that at low temperatures, the CO dissociation barrier is higher than the CO hydrogenation barrier and, consequently, a low CO dissociation rate leads formaldehyde to be the dominant product. Methanol is not formed because formaldehyde adsorption is more favorable than its hydrogenation at low temperatures (T < 600 K), whereas, with a small increase in temperature (from 550 to 600), formaldehyde and CO_2_ compete together as CO dissociation becomes possible, allowing the formation of CO_2_ via the CO + O reaction. As the temperature is increased, the selectivity shifts to ethanol through the coupling reactions of C + CO and CH + CO and the hydrogenation of their products. At even higher temperatures, the methane selectivity increases, and ethylene and formaldehyde appear as side products, which is the reason that the rates of the hydrogenation of C and O are so fast, and the surface coverage is very low. The author’s results also indicated that CH_2_ and CH_3_ hydrogenation as well as CHCH_3_ dehydrogenation are critical reaction steps in controlling the selectivity of products, whereas the formation of hydrocarbons with more than two carbon atoms is negligible since the CH + CH coupling reaction has a higher barrier than that of C + CO coupling. The CO consumption rates were also calculated on the Rh (111) surface but showed much lower values than on the Rh (211) surface. On the Rh (111) surface, methanol formation competes with formaldehyde desorption, as opposed to the Rh (211) surface, where formaldehyde desorption is favored over methanol formation. Hence, the step–edge sites are essential in facilitating CO dissociation to generate a wide range of products (especially ethanol at an intermediate temperature), in addition to formaldehyde and methanol.

**Figure 40:**
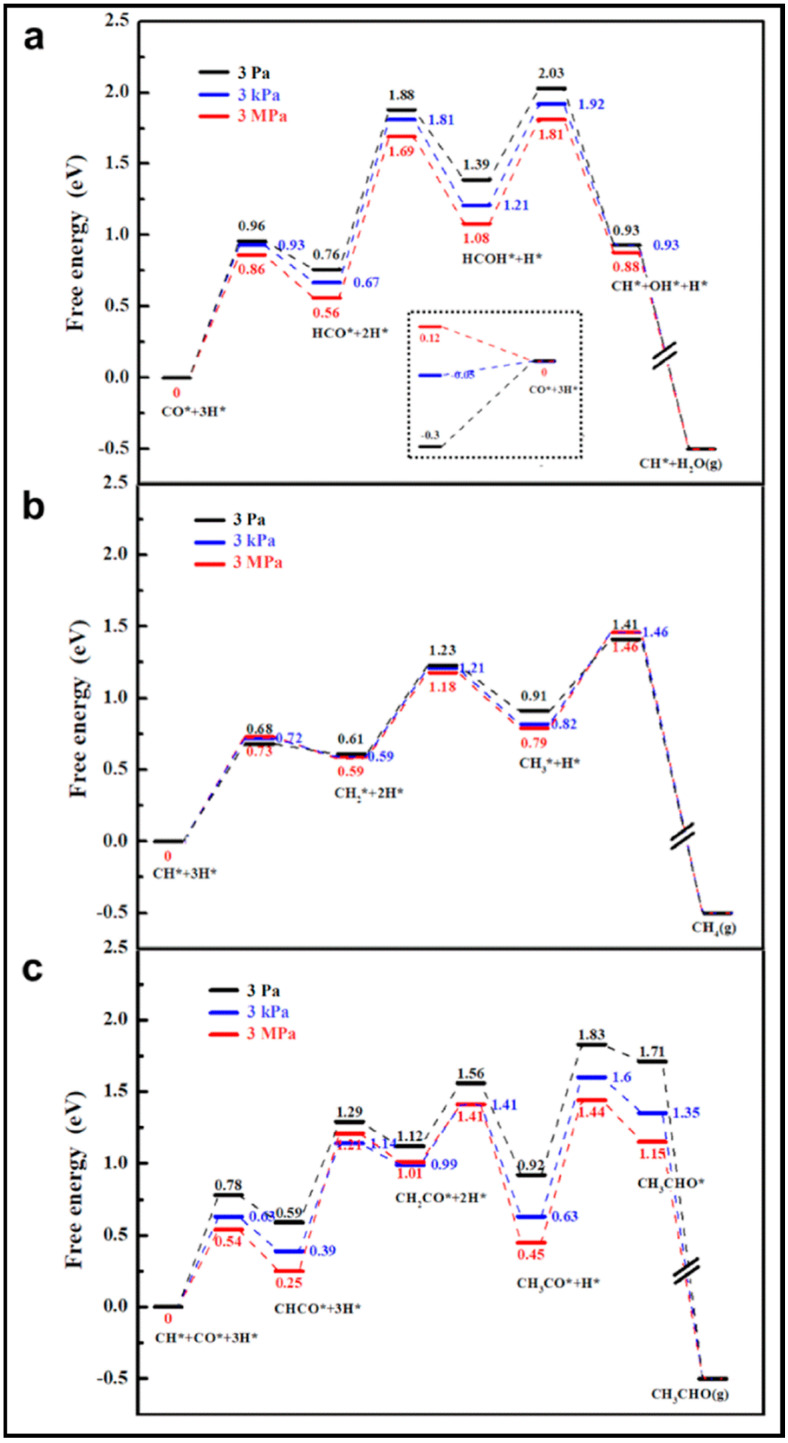
Standard free energy surfaces at three different total pressure of 3 Pa (black line), 3 kPa (blue line), and 3 MPa (red line) for (**a**) CO activation, (**b**) CH_4_ formation, and (**c**) CH_3_CHO formation. The inset of a part denotes the contributions of the adsorption free energy difference between H_2_ and CO [121].

Rh was widely investigated, owing to its selectivity toward C_2+_ oxygenates from syngas (CO + H_2_) conversion. Xu et al. studied the impact of the total pressure variation (PCO/PH_2_) on CO hydrogenation using the Rh (111) surface as the catalyst (See Table 25) [121]. They showed that higher activity could be achieved because of the high surface coverages resulting from an increase in pressure. Table 23 illustrates the remarkable rise in the selectivity toward CH_3_CHO from 0.00% to 83.59%, as the total pressure rises from 3 Pa to 3 MPa. It is expected that the reaction conditions alter the path-controlling reactions. Under 3 kPa, the hydrogenation of CH_3_CO* is the path-controlling reaction in the formation of CH_3_CHO, whereas CH_2_CO* hydrogenation is the path-controlling reaction under 3 MPa. CH_4_ is the only product under 3 Pa in which the production rate and total activity are controlled by CO activation. The free energy surfaces of CO conversion on the Rh (111) surface are shown in Figure 40. The stability trend over increasing pressure from 3 Pa to 3 MPa is similar for CO activation and CH_4_ formation, while the trends for CH_3_CHO production are different. Hence, local coverage controls the activity and selectivity of the products rather than the average coverage.

### 3.5. Bimetallic

**Figure 41:**
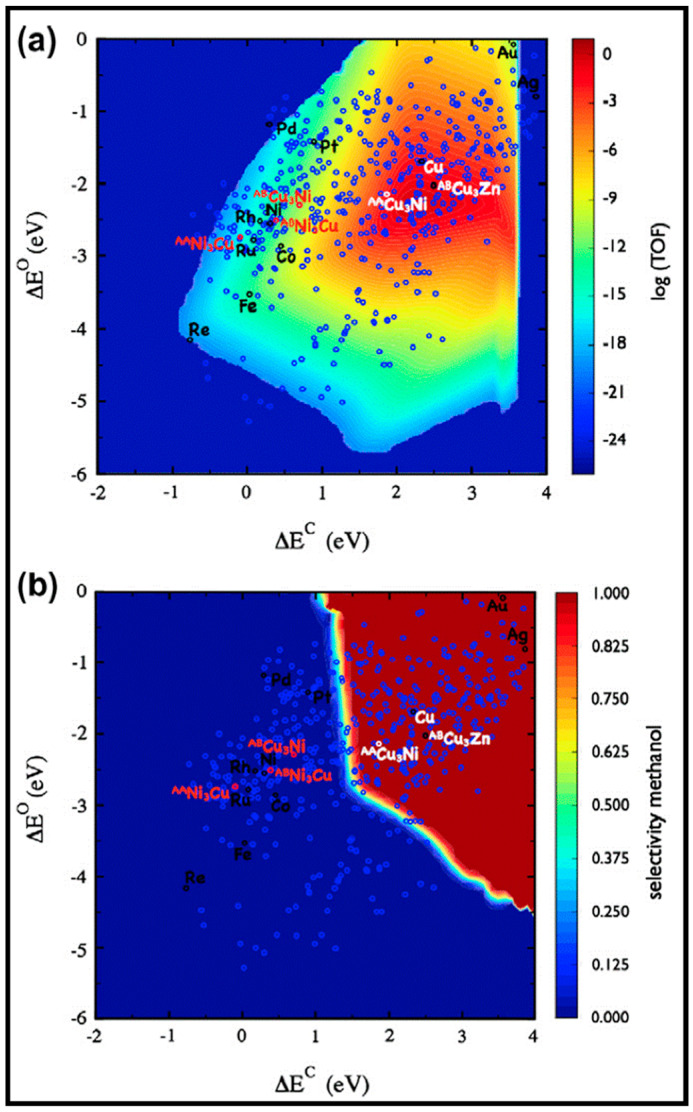
(**a**) Contour plot of activity volcano for methanol formation from CO and H_2_. Small circles indicate the binding energies of carbon and oxygen for binary alloys. (**b**) Selectivity between methane and methanol formation versus the binding energies of carbon and oxygen. Methane formation is calculated under reaction conditions: 523 K, 45 bar H_2_, 45 bar CO, 5 bar CH_4_, and 5 bar H_2_O [122].

A large number of bimetallic alloys, i.e., CuNi alloys that are Cu-rich at steps, were investigated by Studt et al. as potential candidates for active and selective methanol synthesis through CO hydrogenation, Table 26 [122]. First, they considered the stability of the alloys (the type of A_3_B and AB) and chose those with a negative heat of formation as candidate catalysts. The strongest binding site of each alloy is calculated and determined in Figure 41 (blue circles). It was found that many bimetallic alloys are as close as Cu to the top of the volcano. The authors showed that alloying Cu with Zn leads to an increase in the oxygen binding energy and moves Cu toward the top of the volcano. Furthermore, CuNi alloys were revealed to be highly selective for the production of methanol and methane. This is in good agreement with the literature, which reports CuNi catalysts for gas conversion to a mixture of methanol, methane, hydrocarbons, and higher alcohols [108,123], whereas the experimental results also confirmed the methanol selectivity up to 92% for certain preparation methods [123]. CuNi/SiO_2_ and Cu/ZnO/Al_2_O_3_ catalysts were also considered for CO hydrogenation under various conditions, indicating that the main product over a CuNi/SiO_2_ catalyst was methanol. However, Cu/ZnO/Al_2_O_3_ has high activity but less selectivity toward methanol synthesis at higher temperatures (see Table 24).

Recently, methanol synthesis from CO hydrogenation on the defective ZrO_2_-supported In_2_O_3_ (110) surface was investigated in the work of Dou and co-workers [124], who showed that the CO hydrogenation to methanol on the proposed surface is facile, and the rate-determining step is the formation of H_3_CO at a vacancy site [124]. First, H and CO co-adsorb on the surface, eventually forming HCO with an In_3_–C bond. Next, the co-adsorption of HCO and H leads to H_2_CO formation by breaking the In_3_–C bond and stretching the C–H distance. According to the calculated activation barriers, this step is kinetically facile. In the third step, the co-adsorption of H_2_CO and H occurs as H approaches the C atom, and the In_3_–C bond is broken. In this step, the oxygen atom fills the Ov_3_ vacancy site, and an H_3_CO species forms, which is consistent with the reaction energies. Finally, the distance between the O and H atoms stretches, and methanol is produced. As is clear from the relative energies of the elementary steps [124], the H_2_CO + H reaction is the rate-determining step, and the highest reaction barrier in CO hydrogenation is 0.51 eV. Hence, the results indicate that the whole process is energetically favorable, suggesting the applicability of the In_2_O_3_-supported catalyst for CO hydrogenation [124].

In other work, Arab considered CO adsorption on Rh–Cu bimetallic clusters (Rh_x_Cu_4-x_ (x = 0–4)) [80]. They concluded that these clusters are more reactive than pure Cu_4_ for CO adsorption. On all surfaces, the adsorption by the carbon end to the Rh atom(s) is more desirable than by the carbon end to the Cu atom(s) or by the oxygen end. Moreover, the adsorption energies suggest a strong chemical bond between the clusters and CO, except for the Cu_4_-CO-2 and Cu_4_-CO-3 structures, where the carbon is physisorbed. In the case of the Rh_4_ cluster, the higher contribution comes from the d orbitals of Rh and the p orbitals of C, similarly to the Rh_3_Cu-CO-1 structure at the spin multiplicity of 3. The carbon more strongly interacts with the Rh_4_ cluster than the oxygen does. In the Rh_3_Cu-CO-2 structure, the contribution of the s orbitals of the Rh and C atoms is increased, whereas in the Rh_3_Cu-CO-3 structure, one C–Cu bond is created, and the s and p orbitals of the C and Cu atoms play significant roles to form this bond. The adsorption energies confirm the chemical adsorption of CO on the Rh_2_Cu_2_ cluster, whereas the structures of the RhCu_3_, Cu_4_, and Rh_3_Cu clusters are less stable through the increase in the spin multiplicity. Overall, increasing the spin multiplicities causes the stability of Rh-rich systems. It was also found that the CO adsorption at the bridge site requires a large contribution from the d orbitals of the cluster atoms and the p orbitals of C, while, for the CO adsorption on the top sites, the s orbitals of both C and the cluster significantly contribute to the formation of the chemical bonds. Thus, the adsorption at the bridge site was not observed on the RhCu_3_ and Cu_4_ clusters due to the fully occupied d orbitals of the Cu atoms. The maximum adsorption energy is obtained for the Rh_4_ cluster in accordance with the WBI of the CO cluster and in contrast to the WBI of C–O, which confirms the strong interaction between CO and the Rh_4_ cluster and between CO and the Rh-Cu bimetallic clusters compared to the pure Cu_4_ cluster [80]. 

## 4. Summary

This paper was devoted to reviewing the results of investigations on the preferred mechanism of CO dissociation, i.e., the carbide mechanism and the C(H)O insertion mechanism, and of further chain growth. To this end, the activation free energy barriers and the reaction free energies of all elementary reactions were reported for a number of catalysts, and we can report the following general findings:

(i) CO activation is a structure-sensitive reaction. CO activation takes place via three different CO dissociation routes, where the direct route is the most structure-sensitive, particularly on open surfaces, compared to the least structurally sensitive H-assisted routes and CH_x_ hydrogenation. 

(ii) H-assisted CO activation is a favorable route on flat Co surfaces, but both the direct and H-assisted routes are competitive on stepped and defective surface sites. On Fe catalyst surfaces, CO dissociation is inefficient compared to direct CO dissociation, but it is favored at low temperatures and H_2_ pressure. Both direct and H-assisted CO dissociation are feasible on Ni catalyst surfaces, except for the Ni (111) surface, where the preferred pathway is direct dissociation. Hydrogen-assisted pathways are most desirable on flat and stepped Ru catalyst surfaces, but, in contrast, the dominant route for CO dissociation on Rh surfaces is direct CO dissociation. Defect and stepped sites also create a broad range of adsorption sites with different adsorption energies. 

(iii) The chain growth depends on the metal surface, leading to the formation of a wide range of products from methane to heavier hydrocarbons. Methane formation on the flat Co (1000) surface is low, and the presence of defects causes the increasing selectivity toward methane, while the stepped surface suppresses its formation. In contrast with Co, the Fe carbide is superior for the methane selectivity, and Rh is active toward ethanol formation. During the chain-growth process, the CH monomer is the dominant among all the CH_x_ (x = 1–3). The rate-determining steps in the C_1_ and C_2_ hydrocarbon formation are CO hydrogenation to form CHO and CHCH hydrogenation to form CH_2_CH, respectively. Step sites are efficient at facilitating the CHO and CH_2_CH formation and improving the C_2_ hydrocarbon selectivity. 

(iv) Surface coverage is considered a major factor affecting the CO conversion mechanism. CO insertion is preferred at high CO coverage, which increases the activity and selectivity toward C_2_-hydrocarbon formation. Furthermore, lower CH_x_ coverage and, as a consequence, higher H coverage reduce the CO adsorption energy and lower the CO adsorption rate. High H_2_ coverage on the surfaces strengthens the lateral repulsive interactions and plays a key role in determining the CO reactivity. At low-covered surfaces, hydrogen atoms are highly mobile, migrating over the catalyst surface, and it can be inferred that hydrogen adsorption is coverage-dependent. 

(v) Reaction conditions such as temperature and pressure can influence the mechanism governing the FTS process. The selectivity toward various products changes with a variation in temperature, since the activity decreases at low temperatures. Long-chain hydrocarbons and oxygenates are the main products at low temperatures, and methane is the major one at high temperatures. A rise in temperature can also promote direct CO dissociation to become comparable with H-assisted dissociation. Increasing the H_2_ pressure stabilizes the H_2_ adsorption and causes a rise in surface coverage, causing a reduction in the chain-growth probability. 

(vi) O removal through water formation is critical, and feasible OH formation is followed by a rate-limiting OH hydrogenation step.

We trust that this review will be a valuable addition to the literature by collecting a wide variety of computational studies in a single comparison of the efficacies and selectivities of a number of important transition metal catalysts. 

## Figures and Tables

**Table 1:** Calculated elemental reaction barrier and reaction energy (in eV) for CO dissociation (direct and H-assisted pathways) and CH_x_ (x = 0–3) hydrogenation on Co (001), (0001), ($10\bar{1}2$), and ($11\bar{2}0$) surfaces [21,50].

Reactions	(001)		(0001)		$\left(10\bar{1}2\right)$		$\left(11\bar{2}0\right)$	
	Reaction Barrier	Reaction Energy	Reaction Barrier	Reaction Energy	Reaction Barrier	Reaction Energy	Reaction Barrier	Reaction Energy
CO → C + O	-	-	2.46	0.69	1.34	−0.58	1.39	0.32
CO + H → HCO	1.08	0.57	1.18	1.10	1.13	0.36	0.95	0.57
HCO → CH + O	-	-	0.73	−0.80	1.04	−0.91	0.72	−0.54
C + H → CH	-	-	0.73	−0.39	0.69	0.03	0.63	−0.28
CH + H → CH_2_	0.065	0.01	0.55	0.33	0.65	0.52	0.57	0.36
CH_2_ + H → CH_3_	0.96	0.67	0.55	−0.13	0.65	−0.06	0.31	−0.31
CH_3_ + H → CH_4_	1.08	0.49	0.99	−0.07	0.86	0.07	0.76	−0.10

**Table 2:** Activation barrier and reaction energy of elementary reactions in C_2_ hydrocarbon formation on Co (111) surface [14].

	Reaction	E_a_/KJ.mole^−1^	ΔH/KJ.mole^−1^
(R1)	CO → C + O	231.4	89.2
(R2)	CO + H → COH	184.8	88.1
(R3)	CO + H → CHO	130.2	114.1
(R4)	CHO → CH + O	62.9	−63.1
(R5)	CHO + H → CHOH	104.2	25.1
(R6)	CHOH → CH + OH	71.3	−73.5
(R7)	CHO + H → CH_2_O	51.9	13.7
(R8)	CH_2_O → CH_2_ + O	65.3	−54.8
(R9)	CH_2_O + H → CH_2_OH	88.9	31.8
(R10)	CH_2_OH → CH_2_ + OH	54.6	−77.5
(R11)	CH_2_O + H → CH_3_O	46.6	−48.3
(R12)	CH_3_O → CH_3_ + O	142.9	−25.8
(R13)	CH_3_O + H → CH_3_OH	148.6	63.9
(R14)	CH → C + H	101.8	27.6
(R15)	CH + H → CH_2_	55.9	36.0
(R16)	CH_2_ + H → CH_3_	54.2	−11.5
(R17)	CH_3_ + H → CH_4_	91.7	−4.7
(R18)	CH + CO → CHCO	99.0	58.9
(R19)	CH_2_ + CO → CH_2_CO	66.9	60.8
(R20)	CH_3_ + CO → CH_3_CO	132.6	46.1
(R21)	CH + CHO → CHCHO	39.4	19.3
(R22)	CH_2_ + CHO → CH_2_CHO	1.7	0.1
(R23)	CHCO + H → CH_2_CO	61.2	32.9
(R24)	CHCO + H → CHCHO	95.7	68.3
(R25)	CH_2_ CO + H → CH_2_CHO	31.4	−37.3
(R26)	CH_2_CO + H → CH_3_CO	33.2	−33.2
(R27)	CHCHO + H → CH_2_CHO	88.3	−18.9
(R28)	CHCO → CHC + O	138.8	−22.0
(R29)	CH_2_CO → CH_2_C + O	83.6	−48.8
(R30)	CH_3_CO → CH_3_C + O	63.0	−65.4
(R31)	CHCHO → C_2_H_2_ + O	13.0	−127.4
(R32)	CH_2_CHO → CH_2_CH + O	109.4	−17.3
(R33)	CHC + H → CHCH	64.2	−26.5
(R34)	CHC + H → CH_2_C	43.0	−15.7
(R35)	CH_2_C + H → CH_2_CH	0	−0.1
(R36)	CH_2_C + H → CH_3_C	22.3	−46.0
(R37)	CH_3_C + H → CH_3_CH	68.0	58.7
(R38)	CHCH + H → CH_2_CH	107.4	68.2
(R39)	CH_2_CH + H → C_2_H_4_	31.5	−11.6
(R40)	CH_2_CH + H → CH_3_CH	39.1	−4.2
(R41)	CH_3_CH + H → CH_3_CH_2_	44.2	1.7
(R42)	CH_3_CH_2_ + H → C_2_H_6_	61.4	−32.4
(R43)	CH + CH → C_2_H_2_	62.0	−53.4
(R44)	CH_2_ + CH → CH_2_CH	60.8	−15.7
(R45)	CH_2_ + CH_2_ → C_2_H_4_	40.1	−53.0
(R46)	CH_3_ + CH → CH_3_CH	98.4	−2.6
(R47)	CH_3_ + CH_2_ → CH_3_CH_2_	83.5	−23.7
(R48)	CH_3_ + CH_3_ → C_2_H_6_	201.2	−31.8
(R49)	O + H → OH	47.4	16.6
(R50)	OH + H → H_2_O	150.7	60.0
(R51)	OH + OH → H_2_O + O	48.3	4.7

**Table 3:** Activation barrier and reaction energy of rate-determining steps in (KJ·mol^−1^) for C_2_ hydrocarbon formation on flat and stepped Co (111) surfaces [14].

Reaction	Flat Surface		Stepped Surface	
	E_a_	ΔH	E_a_	ΔH
CO + H → CHO	130.2	114.1	117.8	77.1
CHCH + H → CH_2_CH	107.4	68.2	95.7	32.5
CH_3_ + H → CH_4_	91.7	−4.7	97.6	29.7

**Table 4:** Reaction energies (ΔE), dissociation barriers (E_act_), and desorption energies (E_des_) in (kJ/mol) for H_2_ dissociation at different coverages over Co (311), Co (111), and Co (110) surfaces [58].

nH_2_	Surface/Dissociation Route	Co (311)			Co (111)			Co (110)		
		ΔE	E_act_	E_des_	ΔE	E_ads_	E_des_	ΔE	E_ads_	E_des_
1H_2_	1H_2_ → 2H	−51.0	13.0	36.4	−71.1	2.7	21.6	−42.1	14.5	21.6
2H_2_	2H_2_ → 1H_2_ + 2H	−50.2	12.7	37.6	−67.3	4.0	25.8	−40.3	14.2	35.5
	1H_2_ + 2H → 4H	−50.3	11.4	36.8	−53.2	0.6	21.9	−41.7	14.6	33.6
3H_2_	3H_2_ → 2H_2_ + 2H	−45.7	11.7	39.1	−73.3	3.9	20.5	−40.3	14.1	36.9
	2H_2_ + 2H → 1H_2_ + 4H	−51.0	10.6	34.6	−75.3	2.6	26.6	−41.9	13.8	37.0
	1H_2_ + 4H → 6H	−50.0	13.1	35.3	−83.3	5.1	48.7	−34.6	14.7	37.2
4H_2_	4H_2_ → 3H_2_ + 2H	−39.5	11.8	25.3				−40.5	14.8	23.6
	3H_2_ + 2H → 2H_2_ + 4H	−50.0	11.6	19.0				−44.8	12.5	23.7
	2H_2_ + 4H → 1H_2_ + 6H	−53.0	12.6	18.0				−36.0	15.1	26.6
5H_2_	5H_2_ → 4H_2_ + 2H	−62.4	11.9	25.0				−51.2	14.1	23.1
	4H_2_ + 2H → 3H_2_ + 4H							−37.1	13.1	33.8
6H_2_	6H_2_ → 5H_2_ + 2H	−40.4	11.7	26.0				−42.4	13.6	24.3
	5H_2_ + 2H → 4H_2_ + 4H							−50.2	14.3	15.5

**Table 5:** The adsorption energy of carbon monoxide, the distance between the adsorbed hydrogen (atoms or molecules) and cobalt surface Z_H2_, and the distance between the hydrogen atoms in adsorbed hydrogen molecule on the surface (d_H2_) and hydrogen adsorption energy on FCC cobalt surfaces [59].

		111			100			110		
		0.25	0.50	1.00	0.25	0.50	1.00	0.25	0.5	1.00
Adsorption of hydrogen atoms on FCC cobalt surfaces	E_chem_ (KJ/mol)	−344	−334	−262	−371	−349	−299	−360	−338	−279
	Z_H_ (Å)	1.51	1.52	1.56	1.45	1.49	1.52	1.49	1.51	1.54
	ΔH_ads_ (KJ/mol)	−252	−232	−88	−306	−262	−162	−284	−240	−122
Adsorption of hydrogen molecule on FCC cobalt surfaces	E_phys_ (KJ/mol)	−9.6	−7.7	−4.8	−14.4	−12.1	−8.7	−11.6	−8.7	−6.7
	d_H2_ (Å)	0.750	0.748	0.746	0.756	0.754	0.751	0.753	0.751	0.747
	Δd_H2_ (Å)	0.008	0.006	0.004	0.014	0.012	0.009	0.011	0.009	0.005
	Z_H2_ (Å)	1.893	1.895	1.897	7.891	1.892	1.894	1.892	1.894	1.896

**Table 6:** Comparison of the activation energies of main coupling reactions in order that both vertical and horizontal rows show low-to-high adsorption heat of the intermediates [33].

	CH_3_	HCO	HCOH	CH_2_	CH
CH_3_	1.84	0.80	0.77	0.62	0.75
HCO	0.80	_	_	0.05	0.34
HCOH	0.77	_	_	0.16	0.29
CH_2_	0.62	0.05	0.16	0.12	0.49
CH	0.75	0.34	0.29	0.49	0.57

**Table 9:** The activation energies of elementary steps involved in chain propagation and termination for the (left) carbide mechanism and (right) CO insertion mechanism [6].

	Carbide Mechanism				CO Insertion Mechanism		
No.	Elementary Steps	Co (0001)	Stepped Co (0001)	No	Elementary Steps	Co (0001)	Stepped Co (0001)
1	CH_3_ + C → CH_3_C	0.94 ^a^	1.09 ^a^	30	CH + CO → CHCO	1.11 ^e^, 0.99 (0.92)^d^	-
2	CH_3_ + CH → CH_3_CH	1.05 ^a^	1.55 ^a^	31	CH_2_ + CO → CH_2_CO	0.83 ^e^, 0.77 (0.53)^d^	-
3	CH_3_ + CH_2_ → CH_3_CH_2_	1.11 ^a^	0.73 ^a^	32	CH_3_ + CO → CH_3_CO	1.92 ^e^, 1.49 ^d^	1.46 ^f^
4	CH_2_ + C → CH_2_C	0.74 ^a^	1.34 ^a^	33	CH_3_CO → CH_3_C + O	1.30 ^e^, 0.75 (0.92)^d^	-
5	CH_2_ + CH → CH_2_CH	0.76 ^a^	1.32 ^a^	34	CH_2_CO → CH_2_C + O	2.38 ^e^, 0.98 (1.21)^d^	-
6	CH_2_ + CH_2_ → CH_2_CH_2_	0.70 ^a^	0.22 ^a^	35	CHCO → CHC + O	1.87 (1.93) ^d^	-
7	CH + C → CHC	0.91 ^a^	1.96 ^a^	36	CH_2_CO + H → CH_3_CO	1.24 ^e^, 0.78 (0.59) ^d^	-
8	CH + CH → CHCH	0.86 ^a^	1.76 ^a^	37	CH_2_CO + H → CH_2_CHO	0.74 ^e^, 0.87 (0.75) ^d^	-
9	C + C → CC	1.22 ^a^, 0.71 ^c^	2.43 ^a^	38	CHCO + H → CHCHO	1.44 (1.33) ^d^	-
10	CH_3_ + C → CH_3_CH	0.76 (0.60) ^d^	0.86 ^b^	39	CHCO + H → CH_2_CO	1.09 (0.93) ^d^	-
11	CH_3_CH + H → CH_3_CH_2_	-	0.42 ^b^	40	CHCHO + H → CH_2_CHO	0.55 (0.49) ^d^	-
12	CH_3_CH_2_ + H → CH_3_CH_3_	-	0.82 ^b^	41	CH_2_CHO + H → CH_3_CHO	1.41 ^e^, 1.20 (0.92) ^d^	-
13	CH_3_C + C → CH_3_C_2_	-	1..58 ^b^	42	CH_3_CO + H → CH_3_CHO	0.50 ^e^, 0.63 (0.79) ^d^	0.35 ^f^
14	CH_3_C + CH → CH_3_CCH	-	1.44 ^b^	43	CH_3_CHO → CH_3_CH + O	0.52 ^e^, 0.63 (0.73) ^d^	-
15	CH_3_C + CH_2_ → CH_3_CCH_2_	-	1.61 ^b^	44	CH_2_CHO → CH_2_CH + O	1.50 ^e^, 1.37 (1.62) ^d^	-
16	CH_3_CH + C → CH_3_CHC	-	1.28 ^b^	45	CHCHO → CHCH + O	0.73 (1.09) ^d^	-
17	CH_3_CH + CH → CH_4_CHCH	-	1.41 ^b^	46	CH_3_CHO + H → CH_3_CH_2_O	-	0.47 ^f^
18	CH_3_CH + CH_2_ → CH_3_CHCH_2_	-	0.29 ^b^	47	CH_3_CH_2_O + H → CH_3_CH_2_OH	-	1.26 ^f^
19	CH_3_CH_2_ + C → CH_3_CH_2_C	-	1.18 ^b^				
20	CH_3_CH_2_ + CH → CH_3_CH_2_CH	-	1.75 ^b^				
21	CH_3_CH_2_ + CH_2_ → CH_3_CH_2_CH_2_	-	0.74 ^b^				
22	CH_2_C + H→CH_2_CH	0.68 (0.37) ^d^	-				
23	CH_2_C + H → CH_3_C	0.63 (0.28) ^d^	-				
24	CH_2_CH + H → CH_3_CH	0.54 (0.21) ^d^	-				
25	CHC + H → CH_2_C	0.82 (0.70) ^d^	-				
26	CHC + H → CHCH	0.68 (0.57) ^d^	-				
27	CHCH + H → CH_2_CH	1.14 (1.09) ^d^	-				
28	CH_3_CH_2_C + C → CH_3_CH_2_CC	-	1.63 ^b^				
29	CH_3_CH_2_C + CH → CH_3_CH_2_CCH	-	1.47 ^b^				

a [12]. b [79]. c [80].d [63], where the data in the parentheses were calculated on Co (0001) with 1/3 ML CO coverage. e [70]. f [52].

**Table 10:** The activation energies of elementary step for methane formation on various cobalt facets [6].

No.	Elementary Steps	Co (0001)	Stepped Co (0001)	Other Facets
1	C + H → CH	0.41 ^a^, 0.83 ^c^, 0.85 ^d^, 0.73 ^e^	0.77 ^c^, 0.82 ^d^	078 Co_2_ C (001) ^b^, 0.69 Co ($10\bar{1}2$) ^e^, 0.63 Co ($11\bar{2}0$) ^e^
2	C + H → CH_2_	0.37 ^a^, 0.65 ^c^, 0.25, 0.66 ^d^, 0.55, 0.55 ^e^	0.80 ^c^, 0.84 ^d^	078 Co_2_ C (001) ^b^, 0.65 Co ($10\bar{1}2$) ^e^, 0.57 Co ($11\bar{2}0$) ^e^
3	CH_2_ + H → CH_3_	0.60 ^c^, 0.63 ^d^, 0.55 ^e^	0.41 ^c^, 0.43 ^d^	0.43 Co_2_ C (001) ^b^, 065 Co ($10\bar{1}2$) ^e^, 0.31 Co ($11\bar{2}0$) ^e^
4	CH_3_ + H → CH_4_	0.96 ^d^, 1.09 ^d^, 0.99 ^e^	0.88 ^c^, 0.96 ^d^	0.88 Co_2_ C (001) ^b^, 086 Co ($10\bar{1}2$) ^e^, 0.76 Co ($11\bar{2}0$) ^e^

a [72]. b [9]. c [12]. d [75].e [21].

**Table 11:** Activation energy for CO dissociation paths at (left) the B5-B site on Co (221) and (right) the kink site on Co (321) [20].

B5-B Site		Kink Site	
	E_act_ (eV)		E_act_ (eV)
CO → C + O	1.24	CO → C + O	0.91
CO + H → HCO	0.81	CO + H → HCO	0.71
HCO → CH + O	0.49	HCO → CH + O	0.56
CO + H → COH	1.41	CO + H → COH	1.22
COH → C + OH	0.63	COH → C + OH	0.40

**Table 12:** Adsorption energies of the main surface species on the Co-based and Co-promoted catalysts [30,50,85].

Species/ Surface		E_ads_ (eV)			
	Co (001) [45]	Co (111) [30]	Co/Mn/Co (111) [30]	Mn/Co (111) [30]	S/Co (111) [75]
CO	−2.26	−1.76	−2.13	−2.55	−0.72
C	-	−6.95	−7.43	−8.15	−5.82
O	-	−5.73	−6.19	−7.27	−4.67
H	-	−2.84	−3.25	−3.75	−2.71
CH	−7.94	−6.42	−6.83	−7.55	−6.21
CH_2_	−5.61	−4.02	−4.54	−5.44	−3.54
CH_3_	−2.97	−1.93	−2.38	−3.03	−1.212
CH_4_	−0.2	−0.02	−0.02	−0.02	−0.2

**Table 13:** CO_2_/H_2_O ratio generated on Co (0001) and MnO/Co (0001) [19].

CO_2_/H_2_O	503 K	513 K	523 K	533 K
Co (0001)	0.0835	0.1076	0.1254	0.2034
MnO/Co (0001)	0.0158	0.0160	0.0162	0.0196
Ratio of Co (0001) and MnO/Co (0001)	5.28	6.73	7.74	10.38

**Table 14:** Binding energies and Gibbs free energies of reaction for carbon and CH_x_ adsorption on the Co (1 1 1) surface (upper) and stepped Co surface (lower) at surface coverage of 0.25 ML [8].

Species	Binding Energy/ΔG_r_^a^ (KJ/mol C)	
carbon on-surface (hcp hollow)	−658/−4	
subsurface (octahedral)	−660/−6	
CH on-surface (hcp hollow)	−610/−18	
CH_2_ on-surface (hcp hollow)	−400/−17	
Graphene		
carbon at fcc hollow and atop site	−769/−115	
carbon at bridge and near atop site	−770/−116	
Adsorption	Binding energy/ΔG_r_^a^ (kJ/mol carbon)	
	P(4 × 8) unit cell	P(2 × 8) unit cell
Step site (S)	−747/−93	−715/−61
Subsurface (Sub)		−652/+2
P4g clock reconstruction (E1)	−662/−8	−697/−43
Near-edge fcc hollow (E2)		−653/+1

^a^ = Gibbs free energy of reaction for CO (g) + (x/2 + 1) H_2_ (g) M CH*_x_ + H_2_O.

**Table 15:** Carbon deposition energies (eV/C Atom) for flat Co (111), stepped Co (211), and Co (112) surfaces [88].

Number of C Atoms	Co (111) “Flat”	Co (221) “Triangular”	Co (112) “Squared”
1	−0.91	−1.32	−2.06
2	−0.58	−1.26	−1.84
3	−0.97	−1.22	−1.59
4	−1.12	−1.24	−1.34

**Table 16:** The CO adsorption energy (E_ads_), CO dissociation barrier (E_act_), and corresponding reaction energy (ΔE) on various C-rich Co_2_C surfaces [3].

Surfaces	E_ads_ (eV)	E_act_ (eV)	ΔE (eV)	E_f_ (eV)
Window I				
(110)-C_1_A	−1.91	0.83	−1.10	2.23
(110)-C_2_B	−1.70	0.86	−0.92	3.42
Window II				
(111)-C_2_B	−2.02	1.53	−0.38	1.81
(011)-C	−2.11	1.67	0.16	1.09
Window III				
(111)-C_2_A	−2.10	2.11	0.69	0.86
(110)-C_2_A	−1.74	2.13	−0.33	1.26
(110)-C_1_B	−2.02	2.48	−0.29	1.42
(100)-C	−1.96	2.57	0.13	0.90
(101)-C	−2.17	2.65	0.34	1.23
(010)-C	−2.21	3.17	0.36	1.42

**Table 17:** The barriers and the distances of TSs in the water formation on flat Co (0001). Reaction details are as follows: (a) O + H → OH, (b) OH + H → H_2_O, (c) O + H → OH, and (d) OH + H → H_2_O. The distances in b and d show bond lengths between the O in the OH group and the reacting H atom [73].

Reaction	a	b	c	d
Distance (Å)	1.636	1.546	1.516	1.556
Barrier (eV)	1.72	1.42	0.73	1.61

**Table 18:** Relative stabilities of CH_i_ (i = 1–3) and chemisorption energies of C atom, with respect to carbon atom and gaseous CH_4_ on stepped metal surfaces, respectively [99].

	Rh	Co	Ru	Fe
E_1_	0.12	0.07	0.05	0.37
E_2_	0.56	0.66	0.54	1.32
E_3_	0.61	0.43	0.57	1.09
ΔH	−1.00	−0.80	−1.19	−1.40

**Table 19:** Values of E_i,j_ + E_i_ + E_j_, which are summation of coupling reaction barrier and the stability of reactants for the fastest coupling pathways in the C_1_ + C_1_ coupling reactions on stepped metal surfaces [99].

	Rh	Co	Ru	Fe
C + C	2.26	2.46	1.80	2.93
C + CH	1.78	2.04	1.34	2.52
C + CH_2_	2.14	2.02	1.67	2.45
C + CH_3_	2.11	1.55	1.84	2.19
CH + CH	1.68	1.89	1.36	2.79
CH + CH_2_	2.24	2.07	1.84	2.94
CH + CH_3_	2.34	2.08	2.23	2.87
CH_2_ + CH_2_	1.97	1.59	2.00	2.91
CH_2_ + CH_3_	2.04	1.86	2.28	3.20

**Table 20:** Activation barriers of CO dissociation on clean surface with and without hydrogen presence [103].

Reaction	*E*_Forward_ (eV)	*E*_Back_ (eV)	ΔE (eV)
CO ↔ C + O*	1.09	2.02	−0.93
CO ↔ C + O	1.13	2.28	−1.15
CO + 2H ↔ C + O + 2H (asymmetric)	1.06	1.63	−0.57
CO + 2H ↔ C + O + 2H (symmetric)	1.16	1.49	−0.33
CO + 4H ↔ C + O + 4H	0.90	1.15	−0.25

**Table 21:** Activity and selectivity of two iron- and cobalt-based catalysts, 100 Fe/5.1Si/2Cu/3K and 0.5%Pt-25%Co/Al_2_O_3_, at identical conditions: 230 °C, 2.2 MPa, H_2_/CO = 2.1, and 13.0 NL/g-cat/h [107].

Selectivity at 43% CO Conversion			
TOS, h	96.5	140.2	
CH_4_	2.82	8.34	2.95
C_5+_	78.92	83.42	1.06
CO_2_	32.03	0.61	0.02
C_2_ olefin content, %	79.31	8.47	0.11
C_4_ olefin content, %	74.04	54.38	0.73
Market price, USD/LB	0.0245	10.66	435.1

**Table 22:** Activation energies for insertion and migration mechanism compared to the dissociation of carbon monoxide on Ru (0001) [2].

Mechanism	A (cm, mol, s)	Activation Barrier (KJ.mol^−1^)	Activation Barrier (eV)	ΔE (eV)
Desorption				
CO_(s)_ → CO_(g)_	3.5 × 10^13^	189.1	1.96	–
CO Dissociation				
CO_(s)_ → C_(s)_+O_(s)_	3.0 × 10^22^	217.1	2.25	0.69
Insertion				
CH_(s)_+CO_(s)_ → CHCO_(s)_	3.7 × 10^21^	109.9	1.14	0.65
C〖H_2_〗_(s)_+CO_(s)_ → CH_2_ CO_(s)_	3.7 × 10^21^	116.8	1.21	0.47
Migration				
CH_(s)_+CO_(s)_ → CHCO_(s)_	3.7 × 10^21^	116.8	1.21	0.56
C〖H_2_〗_(s)_+CO_(s)_ → CH_2_ CO_(s)_	3.7 × 10^21^	144.7	1.50	0.42
Carbonyl Dissociation				
CHCO_(s)_ → CHC_(s)_+O_(s)_	3.0 × 10^22^	201.6	2.09	0.35
CH_2_CO_(s)_ → CH_2_ C_(s)_+O_(s)_	3.0 × 10^22^	137.9	1.43	0.35

All reactants are located in the hcp position.

**Table 23:** Elementary steps and related reaction enthalpies and activation energies for (A) H*-assisted and (B) direct CO* activation paths on the (111) terrace of Ru (201) (1.55 ML CO*) [116].

(A)	**CO Activation Pass**	**Step**		**Energy (KJ mol^−1^)**
	H*-assisted	**1.** ${CO}_{\left(g\right)}+*$ 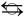 ${CO}^{*}$	ΔH_1_	−42
		2. $H_{2}+*$ 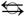 $2H^{*}$	ΔH_2_	−15
		3. ${CO}^{*}+H^{*}$ 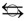 $HCO^{*}+*$	ΔH_3_	50
		4. $HCO^{*}+H^{*}$ 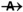 $HCOH^{*}+{\;}^{*}$	E_4_	88
			E_app_ = ΔH_2_+ ΔH_3_+ E_4_ − ΔH_1_	165
(B)		Step		Energy (KJ mol^−1^)
	Direct	1.CO (g) + * 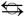 CO*	ΔH_1_	−42
		6.CO* + * 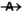 C* + O*	E_6_	280
			E_app_ = E_6_ − ΔH_1_	322

**Table 24:** Rate constants of chain-propagation and chain-growth termination at T = 220 °C for the most preferred routes in both carbide and CO insertion mechanism [119].

Route	Mechanism	k_p_ (mol S^−1^)	k_t_ (mol S^−1^)
CHCH	Carbide	6.79 × 10^2^	5.88 × 10^−4^
CCH_3_	Carbide	6.79 × 10^−1^	5.88 × 10^−4^
CH_2_CH_2_	Carbide	6.79 × 10^−2^	5.88 × 10^−4^
CHCO	CO insertion	6.79 × 10^−8^	5.88 × 10^−4^
CHCHO	CO insertion	6.79 × 10^−12^	5.88 × 10^−4^
CH_2_CO	CO insertion	6.79 × 10^−13^	5.88 × 10^−4^
CH_2_CHO	CO insertion	6.79 × 10^−17^	5.88 × 10^−4^

**Table 25:** Selectivity toward CH_3_CHO and CH_4_ in CO conversion on Rh (111) surface under different pressures [121].

Pressure	CH_3_CHO Selectivity (%)	CH_4_ Selectivity (%)
3 Pa	0.00	100.00
3 kPa	2.47	97.53
3 MPa	83.59	16.41

**Table 26:** The selectivity, TOF, etc., of CuNi/SiO_2_ and Cu/ZnO/Al_2_O_3_ catalysts during CO hydrogenation at steady-state condition of 100 bar, and H_2_/CO = 1.0 *v*/*v* [122].

Catalysts	T (°C)	GHSV (h^−1^)	*X*_CO_ (mol%)	Carbon-Based, CO_2_-Free Selectivity (mol%)			MeOH_STY_ g/(kg_cat_ h)	TOF ^a^ mol_MeOH_ m^−2^ s^−1^
				MeOH	C_2+_ Oxygenates	HC		
CuNi/SiO_2_	250	2000	5.2	99.2	0.4	0.4	65	6.7 × 10^−5^
CuNi/SiO_2_	275	2000	12.1	99	0.4	0.6	167	1.7 × 10^−4^
CuNi/SiO_2_	300	4160	11.2	99	0.5	0.5	330	3.4 × 10^−4^
Cu/ZnO/Al_2_O_3_	250	16,000	7.2	99	0.9	0.1	842	9.2 × 10^−5^
Cu/ZnO/Al_2_O_3_	275	16,000	13.5	97.6	2.1	0.3	1315	1.4 × 10^−4^
Cu/ZnO/Al_2_O_3_	300	32,000	12.7	96.3	2.8	0.9	2666	2.9 × 10^−4^

^a^ Molar amount of methanol based on active surface area of catalysts.

## Data Availability

No new data created in this study.
